# Chromosomal passenger complex condensates generate parallel microtubule bundles *in vitro*

**DOI:** 10.1016/j.jbc.2024.105669

**Published:** 2024-01-23

**Authors:** Ewa Niedzialkowska, Tan M. Truong, Luke A. Eldredge, Aamir Ali, Stefanie Redemann, P. Todd Stukenberg

**Affiliations:** 1 Department of Biochemistry and Molecular Biology, University of Virginia School of Medicine, Charlottesville, Virginia, USA; 2 Department of Cell Biology, University of Virginia School of Medicine, Charlottesville, Virginia, USA; 3 Department of Molecular Physiology and Biological Physics, University of Virginia School of Medicine, Charlottesville, Virginia, USA; 4 Center for Membrane and Cell Physiology, University of Virginia School of Medicine, Charlottesville, Virginia, USA

**Keywords:** mitotic spindle, midzone, inner centromere, Aurora kinase, chromosomal instability, MTOC, chromosomal passenger complex

## Abstract

The mitotic spindle contains many bundles of microtubules (MTs) including midzones and kinetochore fibers, but little is known about how bundled structures are formed. Here, we show that the chromosomal passenger complex (CPC) purified from *Escherichia coli* undergoes liquid–liquid demixing *in vitro*. An emergent property of the resultant condensates is to generate parallel MT bundles when incubated with free tubulin and GTP *in vitro*. We demonstrate that MT bundles emerge from CPC droplets with protruding minus ends that then grow into long and tapered MT structures. During this growth, we found that the CPC in these condensates apparently reorganize to coat and bundle the resulting MT structures. CPC mutants attenuated for liquid–liquid demixing or MT binding prevented the generation of parallel MT bundles *in vitro* and reduced the number of MTs present at spindle midzones in HeLa cells. Our data demonstrate that an *in vitro* biochemical activity to produce MT bundles emerges after the concentration of the CPC and provides models for how cells generate parallel-bundled MT structures that are important for the assembly of the mitotic spindle. Moreover, these data suggest that cells contain MT-organizing centers that generate MT bundles that emerge with the opposite polarity from centrosomes.

During anaphase, microtubule (MT) structures form in the central region of the spindle known as the spindle midzone (hereafter midzone), which comprises parallel-bundled MTs linked by antiparallel cross-linked plus ends to define the cytokinetic furrow at the cell cortex (1, 2). MT bundled structures also emerge under the cortex at the region forming the cytokinetic furrow (3). Midzones have key functions in anaphase, such as contributing to anaphase B movements of chromosomes by generating a force that pushes poles apart (4) and in generating a signal that defines the location of the cytokinetic furrow (5). Midzones are composed of sets of bundled MTs, with each unit having two parallel bundled MTs oriented toward the center of the spindle where they are joined by the protein regulator of cytokinesis 1, which has an antiparallel cross-linking activity. Additional regulators include Kif4a, which is a kinesin that caps MT plus ends (6, 7), and the cytoplasmic linker-associated proteins that together regulate the stabilization, dynamics, and formation of midzone MTs (8). Finally, there is a set of proteins required to generate the signal for the generation of the cytokinetic furrow known as the centralspindlin complex (9).

The chromosomal passenger complex (CPC) is also localized to midzone, where it generates gradients of kinase activity that provide spatial information for anaphase events (10, 11, 12). The CPC contains Aurora kinase B and is a critical regulator of several stages of mitosis. The CPC shows dynamic localization throughout mitosis as it moves from chromosomes in prophase, to inner centromeres in prometaphase, to the midzone during the metaphase to anaphase transition (13, 14) and finally becoming a midbody component. The CPC is required for cytokinesis, but it is unclear if this is a function of the midzone pool or another pool that forms at the plasma membrane at the presumptive furrow in anaphase (15, 16, 17). The CPC is a heterotetrameric assemblage of the scaffolding protein, INCENP, that binds and regulates the Aurora kinase B at its C terminus, whereas its N-terminal coiled-coil binds to both the chromatin-binding Survivin and phase-separating Borealin subunits (18).

The activation of Aurora kinase B activity is stimulated by MTs *in vitro* and in *Xenopus* extracts (19, 20, 21). The CPC contains at least two MT-binding sites: one within the N terminus of Borealin (22) and the other a single α-helix (SAH) domain of INCENP (23). It is unclear, however, whether the CPC functions are limited to regulating and localizing Aurora kinase B signaling or there exists additional roles pertaining to organizing MTs in the midzone. It has recently been shown that the centromere-targeting region of the CPC, which is composed of Survivin, Borealin, and the N terminus of INCENP (hereafter referred to as the CEN domain), binds and bundles MTs *in vitro*. This property is thought to be mediated by the Borealin subunit, which contains an N-terminal MT-binding site (16) and a central intrinsically disordered domain that drives liquid–liquid demixing (LLD) of CPC subassemblies *in vitro* (24). LLD is driven by weak multivalent interactions and can result in high concentrations of proteins that may impart new biochemical properties that are not found in soluble proteins. LLD has been proposed to be an important driver of many MT-based activities. The pericentriollar region may be a nonmembranous organelle, and its ability to concentrate tubulin and sequester the γ-TuRC complex may underlie its ability to nucleate MTs (25, 26). Similarly, branched MT structures of the mitotic spindle require LLD activity of the TPX2 protein that can concentrate free tubulin (27). Furthermore, the importance of the CPC LLD and MT bundling activity in anaphase is unclear, but the amount of the CPC in midzones is reduced in mutants deficient in CPC LLD (24), and the complex does not bind to the central spindle or the midbody if mutated in the MT-binding region of Borealin (28). It is currently controversial if the CPC undergoes LLD at centromeres, but even if it does not, the formation of CPC condensates *in vitro* provides an experimental opportunity to determine if new biochemical activities arise from concentrating the CPC in the inner centromere or on midzone MTs.

Here, we identify an emergent *in vitro* property that arises from combining the CPCs *in vitro* activities to bind MTs, to liquid–liquid demix, and to sequester free tubulin in condensates. We have expressed and purified tetrameric CPC and demonstrate its ability to undergo LLD *in vitro*. CPC condensates concentrate free tubulin which, in the presence of GTP, undergo polymerization and the resulting MTs organize into MT bundles. These bundles emerge from CPC condensates with uniform polarity, extending their minus ends distally, which is the opposite polarity to other MT-organizing centers (MTOCs), such as centrosomes and Golgi apparatus. Finally, we provide evidence that these previously unappreciated biochemical activities of the CPC are important in cells, as midzone formation and cortical bundles of MTs are deficient in cells expressing CPC mutants deficient in either MT bundling or LLD. Altogether, our data indicate that the concentration of the CPC at centromeres and midzones can generate new emergent biochemical properties. Moreover, to our knowledge, the CPC is the first complex to contain an *in vitro* activity to generate MT structures that grow from an organizing structure in the opposite polarity of centrosomes and other MTOCs.

## Results

### The CPC undergoes LLD *in vitro*

**Figure 1:**
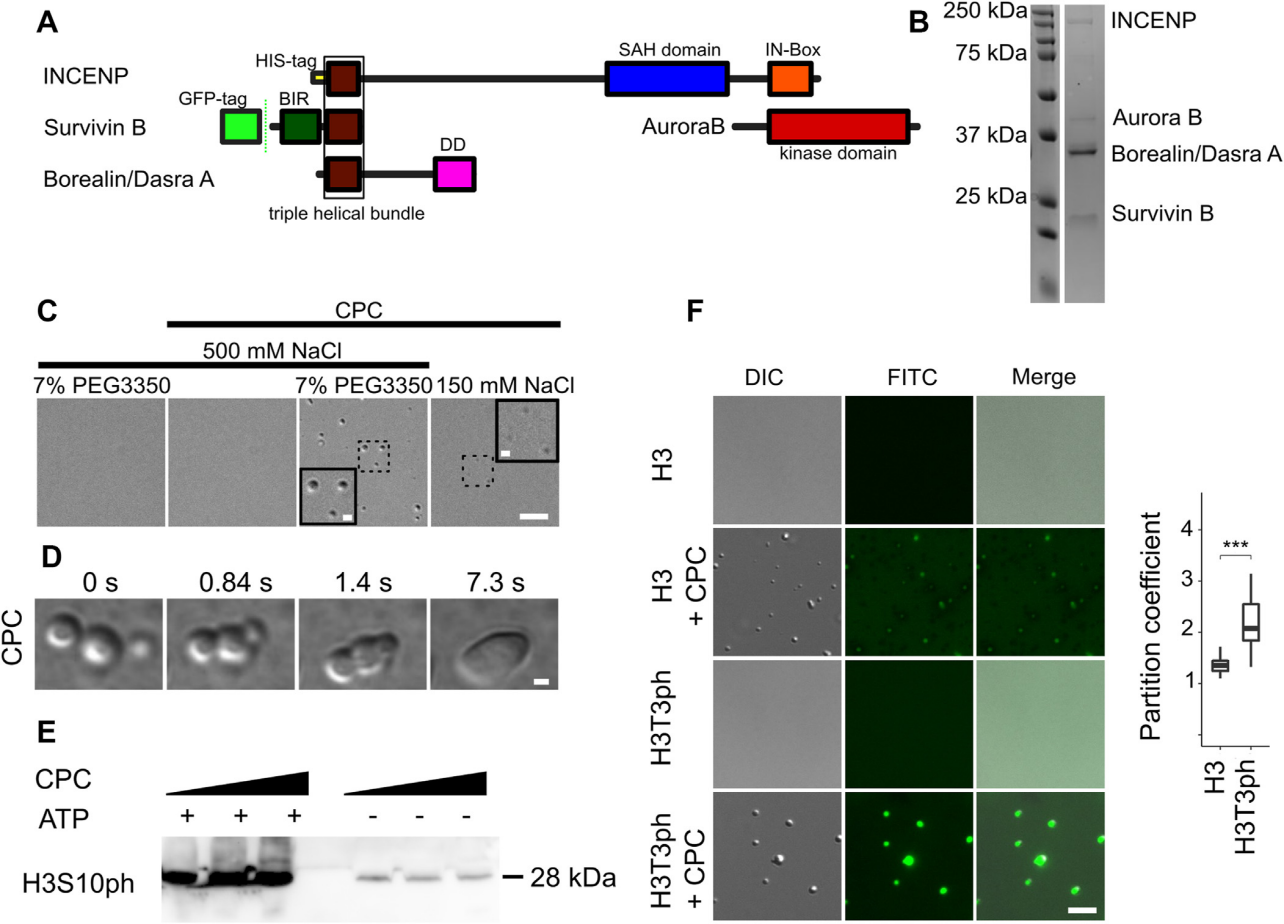
**Recombinant CPC undergoes liquid–liquid phase separation.***A*, scheme of the CPC; BIR: baculovirus IAP repeat (*dark green*); SAH: single α-helix domain (*blue*); IN-Box: INCENP conserved box (*red*); DD—dimerization domain (*magenta*); triple helical bundle composed of the N terminus of Borealin and INCENP and the C terminus of Survivin (*brown*); Aurora kinase B domain (*dark red*). *B*, Coomassie-stained SDS-PAGE of purified recombinant CPC. *C*, differential interference contrast (DIC) of CPC condensates; scale bar represents 5 μm. *Insets* showed magnified views of *dotted regions*; scale bar represents 1 μm. *D*, DIC images demonstrating fusion of CPC coacervates; scale bar represents 1 μm. *E*, phase-separated CPC contains active kinase as measured by H3S10ph immunoblot after incubation with histone H3 Ser10 and ATP in the presence of 0.25, 0.5, and 1 μM LLPS CPC. *F*, phase-separated CPC retains the ability to specifically bind H3T3ph peptides. DIC and fluorescence images showing partitioning of histone H3T3ph (1–21)-FITC and H3T3 (1–21)-FITC into CPC condensates. Plot to the *right* shows calculated partition coefficient of H3T3ph (1–21)-FITC peptide (n = 42) and H3T3 (1–21)-FITC peptide (n = 42) into coacervates. Experiment was repeated twice; *p* = 2.2 × 10^−16^; scale bar represents 5 μm. Statistical analysis was performed by applying Kolmogorov–Smirnov test, ∗*p* < 0.05, ∗∗*p* < 0.01, ∗∗∗*p* < 0.001. Box and whisker graph represents the median (*central line*), 25th to 75th percentile (*bounds of the box*), and 5th to 95th percentile (*whiskers*). DIC, differential interference contrast; LLPS, liquid–liquid phase separation.

The *Xenopus laevis* CPC (Fig. 1, *A* and *B*) was expressed in *Escherichia coli* and purified on nickel–nitrilotriacetic acid resin *via* a 6xHIS tag fused to the N-terminus of INCENP. The purified complex migrated on size-exclusion chromatography as a complex under high salt conditions (Fig. S1*A*). The catalytic component of the complex, Aurora kinase B, autoactivates during bacterial expression and retains high activity throughout purification, including toward other members of the complex. The stoichiometry and identity of all four subunits of the CPC were confirmed by mass spectroscopy analysis (Fig. S1*B*). These analyses suggest that the preps employed in this study contain approximately twofold higher amounts of the Borealin subunit compared with Aurora B and full-length INCENP, most likely because of some proteolysis of the INCENP subunit. A disordered region of Borealin (amino acid residues 139–160) is involved in LLD of a subcomplex of the CPC (24). Recombinant CPC undergoes LLD *in vitro* in the presence of a crowding agent (7% PEG3350) or in a low salt buffer, as in these conditions, we observed the formation of spherical droplets characteristic of LLD (Fig. 1*C*). These droplets fused at early time points after inducing phase separation, demonstrating their liquid properties (Fig. 1*D*). All the CPC components are represented in the coacervates upon phase separation, as shown by pelleting assays (Fig. S1*C*). The CPC droplets formed at 1 μM, which is below the concentration that has been estimated for Aurora B at centromeres (29). *In vitro* CPC droplets retained specific binding to histone H3 peptide phosphorylated on T3 and Aurora kinase B activity (Fig. 1, *E* and *F*).

### The CPC nucleates and bundles MTs

**Figure 2:**
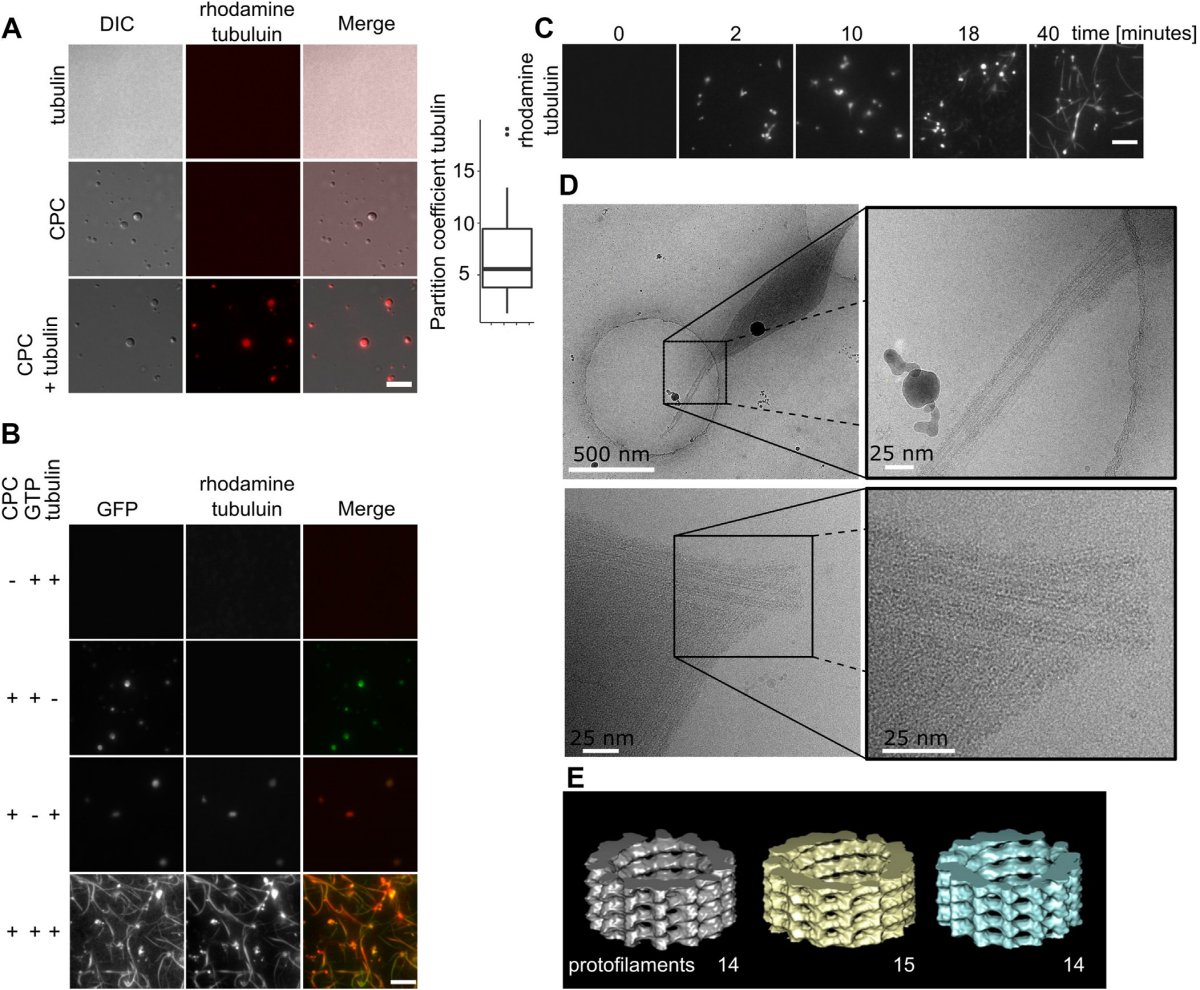
**CPC coacervates generate bundles of MTs.***A*, CPC condensates concentrate free tubulin. DIC and fluorescence images of rhodamine-labeled tubulin dimers partitioning into CPC coacervates (n = 43). Experiment was repeated multiple times during the course of the project using different CPC preps (n = 3 preps). Box and whisker graph represents the median (*central line*), 25th to 75th percentile (*bounds of the box*), and 5th to 95th percentile (*whiskers*). *B*, CPC condensates generate tapered MT bundles in the presence of GTP. GFP-CPC was incubated with rhodamine-labeled tubulin and GTP as indicated. Experiment was repeated twice. *C*, bundles of MTs generated in the presence of phase-separated CPC imaged by TIRF microscopy after the indicated time. Experiment was repeated multiple times during the course of the project using three different CPC preps; scale bar for *A*, *B*, and *C* represents 5 μm. *D*, cryo-EM images of bundles of MTs generated by the CPC. *E*, 3D reconstruction of single MTs generated by the CPC. Cryo-EM measurements were done twice from two independent CPC preps. CPC, chromosomal passenger complex; DIC, differential interference contrast; MT, microtubule; TIRF, total internal reflection fluorescence.

The CPC regulates assembly of chromatin-associated MTs by controlling the activity of the MT depolymerase, MCAK (30, 31) *in vivo*. However, it is unclear if the CPC alone can directly regulate MT formation. We previously showed that α/β-tubulin dimers are enriched in condensates of a CPC subcomplex and that mixing these condensates with tubulin in the presence of GTP generated weak elongated structures (24). Here, we tested whether the full-length CPC concentrates free tubulin and promotes MT polymerization. α/β-tubulin dimers were approximately fivefold enriched in CPC condensates (Fig. 2*A*). As a control, GFP showed no preferable enrichment in condensates compared with the dilute phase (Fig. S2*A*). When CPC coacervates were incubated with free tubulin in the presence of GTP, elongated structures were observed within 10 min of incubation (Fig. 2*C*). No MT structures were formed in the absence of GTP or CPC (Figs. 2*B* and S2*B*), and GMpCpp-polymerized MTs incubated with PEG3350 did not bundle (Fig. S2*C*). We imaged the resulting structures by cryo-EM, which confirmed that the elongated structures were composed of tightly packed MT bundles (Fig. 2*D*). We performed 3D reconstruction on three single MTs, although the tight packing of MTs within a bundle made it difficult to definitively select a greater number of single MTs for reconstruction. Those three single MTs generated by the CPC were composed of 14 or 15 protofilaments (Fig. 2*E*). Polymerized MTs formed bundles, and no astral-like MTs were observed in any of EM images. The MT bundles were coated with unstructured material. Based on fluorescence microscopy images showing colocalization of MTs and CPC (Fig. 2*B*), we posit this material to be CPC. Taken together, these results show that phase separation of CPC concentrates free tubulin and drive MT polymerization and bundling *in vitro*.

### MTs promote CPC liquid–liquid phase separation

**Figure 3:**
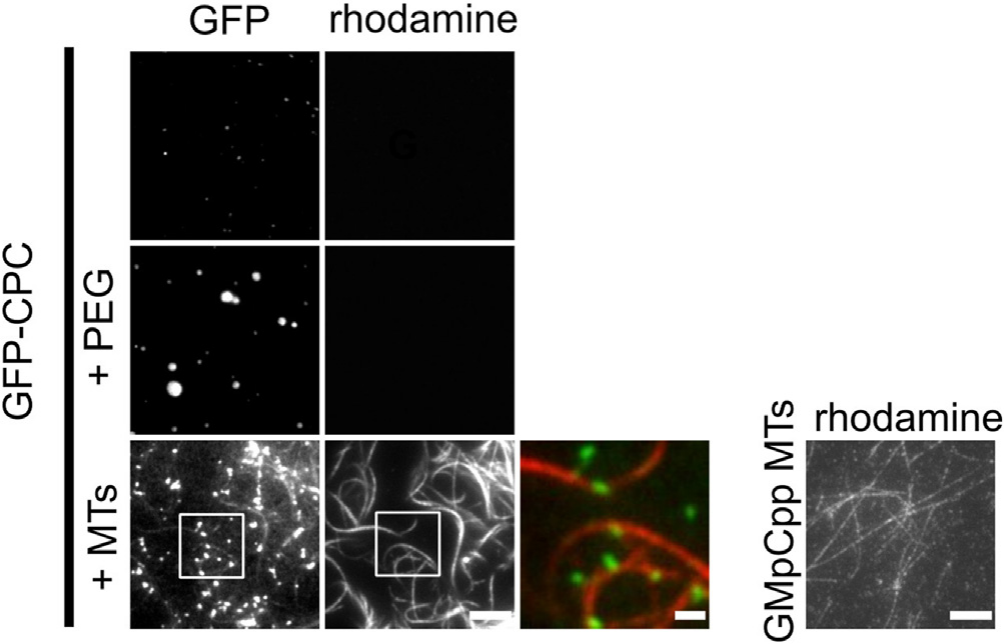
**MTs induce phase separation of CPC.** About 100 nM of GMpCpp-stabilized rhodamine-labeled MTs induced phase separation of 1 μM soluble GFP-CPC as imaged by TIRF microscopy; scale bar for *gray scale* images represent 5 μm; scale bar for pseudo-colored *inset* represents 1 μm. The *top panel* shows soluble GFP-CPC/CPC sample; *middle panel* shows CPC phase separation induced by PEG; the *bottom panel* shows CPC phase separation induced by MTs (conditions without PEG). For reference, image to the *right* shows GMpCpp-stabilized rhodamine-labeled single MTs (not bundled) before the incubation with the CPC; scale bar represents 5 μm. Experiment was repeated twice. CPC, chromosomal passenger complex; MT, microtubule; TIRF, total internal reflection fluorescence.

α-satellite DNA, chromatin, or MT bundles induce phase separation of a CEN domain of the CPC. Similarly, free tubulin enhances the formation of TPX2 condensates (25). We investigated whether MTs can influence the CPC condensation state by mixing GFP-CPC at a concentration near the saturation concentration (Fig. 3, *top of the panel*) with GMpCpp-stabilized MTs and visualized the formation of condensates by fluorescence and differential interference contrast (DIC) microscopy (*bottom of the panel*). The addition of MTs reduced the saturation concentration as visualized by the increase of CPC signal in condensates (Fig. 3). We conclude that MTs promote phase separation of the CPC.

### Polarity of bundles of MTs generated by the CPC

**Figure 4:**
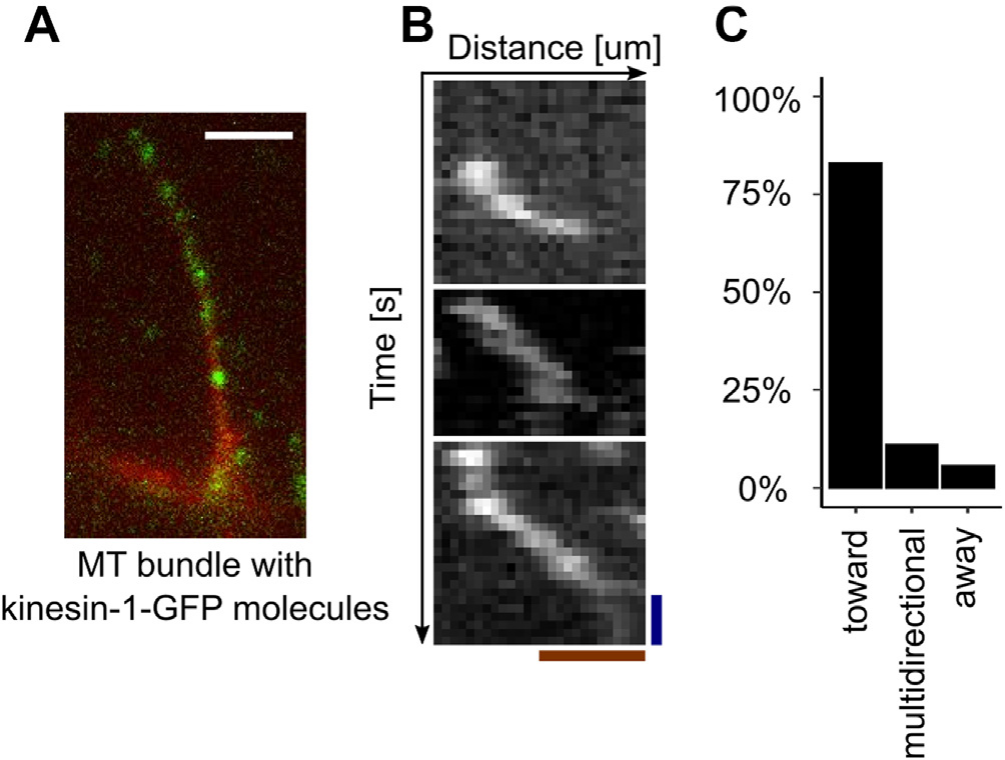
**MTs generated by LLPS CPC are oriented with plus ends inside the CPC condensate.***A*, an example of single Cy5-labeled MT bundle with kinesin-GFP molecules (*green dots*); scale bar represents 5 μm. Movie S1 shows the movement of the kinesins toward the CPC droplet (at the *bottom of the image*). *B*, representative kymographs (from two independent experiments with 92 tracks) of kinesin-1-GFP molecules moving on bundle of MTs generated by the CPC. Scale bar represents *blue*—1 s and *red*—1 μm. *C*, classification of MT bundles based on predominant direction of kinesin-1-GFP movements relative to CPC condensate: toward (defined as ≥75% of recorded kinesin movements on given bundle toward CPC condensate), or away (≥75% movements away from CPC condensate), or in both directions (multidirectional, remaining bundles). CPC, chromosomal passenger complex; LLPS, liquid–liquid phase separation; MT, microtubule.

To understand the polarity of MTs protruding from CPC coacervates, we added purified kinesin-1-GFP (Fig. S4*A*), a plus-end directed motor, to MT bundles generated by the CPC and followed the direction of kinesin-1 movements by total internal reflection fluorescence (TIRF) microscopy (Fig. 4, *A* and *B* and Movie S1), a technique known as motor-PAINT (32). We compared the direction of movements on a single bundle to determine if all the MTs had a similar orientation. We grouped bundles based on direction of kinesin-1 movements into three classes; toward or away from CPC condensate, or multidirectional (Fig. S4*B*). Kinesin-1 movements on the majority (72%) of the bundles were uniformly oriented toward the CPC condensate, whereas a further 11% of bundles analyzed displayed ≥75% of movements toward condensates (Fig. 4*C*). These findings suggest that the CPC-generated bundles of polymerized MTs are composed of parallel MTs, and that these are oriented with their plus ends inside or on the surface of CPC coacervates. These results suggest that MTs generated by CPC coacervates are parallel bundles oriented with plus ends adjacent to or inside the nucleating condensate.

### Borealin’s phase separation and MT-binding activities are required to assemble MT bundles

**Figure 5:**
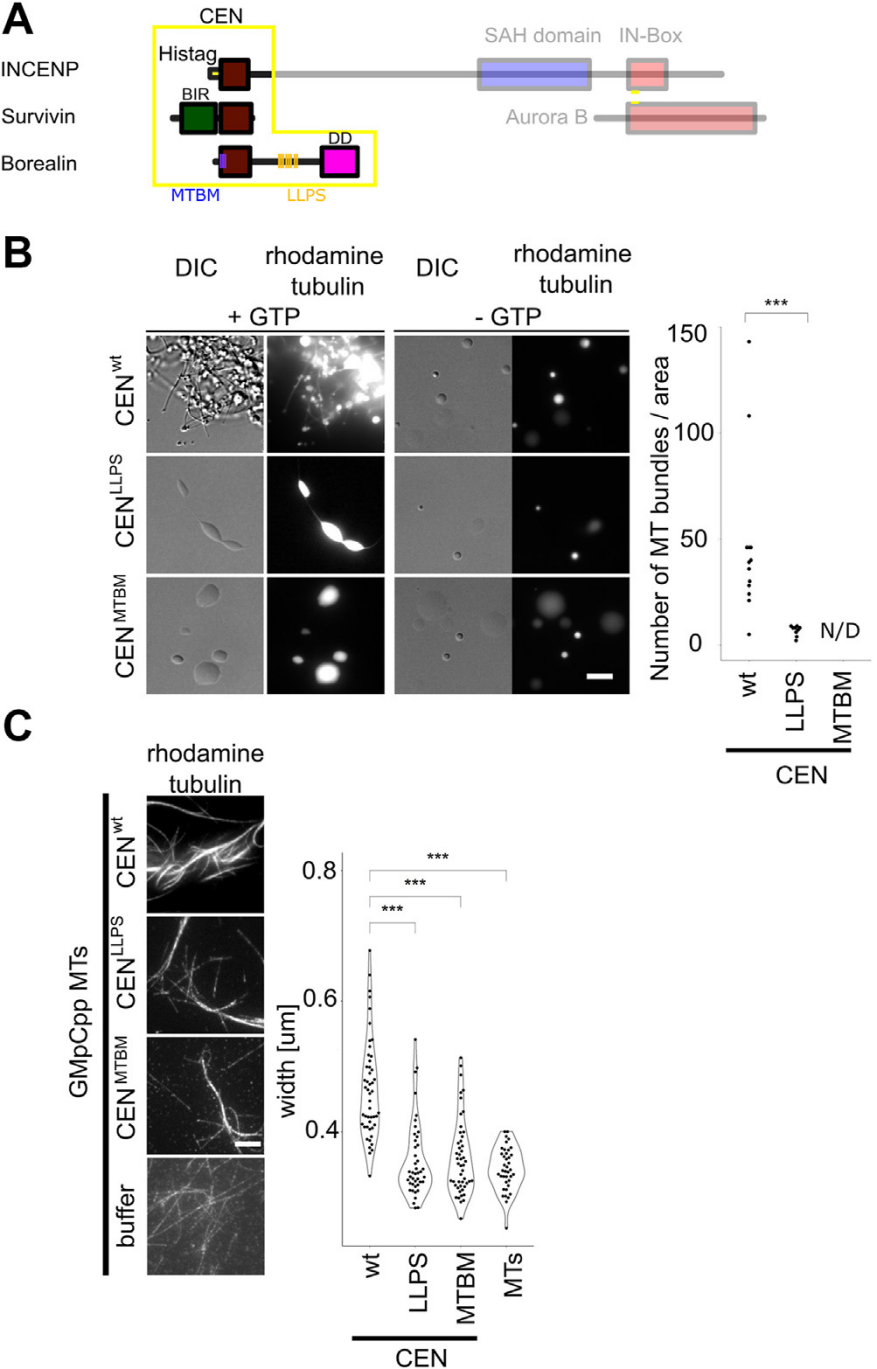
**Phase separation propensity and MT binding of Borealin subunit of the CPC are important for MT nucleation and bundling.***A*, scheme of the CPC; CEN: centromere-targeting domain composed of full-length Survivin and Borealin, N-terminal fragment of INCENP. Mutations introduced to Borealin amino acid sequence are presented as *blue* (MTBM) and *orange* (LLPS) lines. The regions of the CPC that are not part of the CEN domain are faded out. *B*, DIC and fluorescence images of rhodamine-labeled MTs generated by CEN-Borealin^wt^, CEN-Borealin^LLPS^, and CEN-Borealin^MTBM^. Experiment was repeated three times. Plot shows the number of MT bundles per μm^2^; total number of MT bundles for CEN-Borealin^wt^ (n = 612), for CEN-Borealin^LLPS^ (n = 61); *p*^wt-LLPS^ = 5.5 × 10^−5^, CEN-Borealin^MTBM^—not detected (N/D); scale bar represents 5 μm. *C*, GMpCpp-stabilized rhodamine-labeled MT bundled by CEN-Borealin^wt^, CEN-Borealin^LLPS^, and CEN-Borealin^MTBM^ imaged in TIRF mode with excitation at 555 nm (rhodamine tubulin). Plot shows quantification of the width of single bundles of MTs. Experiment was repeated twice; CEN-Borealin^wt^ (n = 49), CEN-Borealin^LLPS^ (n = 43), CEN-Borealin^MTBM^ (n = 49), MTs (n = 39); *p*^wt-LLPS^ = 1.05 × 10^−9^; *p*^wt-MTBM^ = 1.68 ×10^−9^. Statistical analysis was performed by applying Kolmogorov–Smirnov test; ∗*p* < 0.05, ∗∗*p* < 0.01, ∗∗∗*p* < 0.001; scale bar represents 5 μm. CPC, chromosomal passenger complex; DIC, differential interference contrast; LLPS, liquid–liquid phase separation; MT, microtubule; TIRF, total internal reflection fluorescence.

We were unable to purify human CPC from *E. coli* after multiple attempts; so we moved to a subassembly of human CPC components to identify mutants that can test if the MT-bundling activity of condensates has a role during mitosis in human cells. This subassembly, which we call the CEN subcomplex (previously called ISB (24)) because it contains all the activities required to localize the CPC to the centromere, contains full-length Survivin and Borealin and the first 54 amino acids of INCENP. The CEN subcomplex was sufficient to form robust MT bundles demonstrating that the activity is conserved from *Xenopus* to humans and independent of kinase activity and the MT binding activity on the SAH domain of INCENP (Fig. 5*A*).

To identify mutants to test the importance of the MT-bundling activities in human cells, we characterized a set of human mutant CPC CEN subcomplexes (Figs. 5*A* and S5*A*). To determine whether Borealin phase separation was important for MT nucleation and bundling, we employed a CEN mutant where five lysines and three arginines within a 21 amino acid stretch of an intrinsically disordered region of the Borealin subunit are mutated to alanines (Borealin^R141A, K143A, R144A, K149A, K150A, R151A, K158A, K160A^; CEN-Borealin^LLPS^), which has a reduced tendency to undergo phase separation compared with the WT protein (Fig. S5*C*) (24). All components of the CEN WT, CEN-Borealin^LLPS^, remained in the coacervates upon phase separation as shown by pelleting assays (Fig. S5*B*). The condensates of CEN-Borealin^LLPS^ that did form generated significantly fewer MTs than condensates of the CEN-Borealin^wt^ (Fig. 5*B*).

Next, we checked the role of the MT-binding region of Borealin in the formation of MT bundles. We used a charge-reversed CEN-Borealin^R17E,R19E,K20E^ mutant that has reduced MT-binding affinity (CEN-Borealin^MTBM^) (22). The Borealin MTBM mutant was previously characterized in cells, and it was demonstrated that adding an exogenous MT-binding domain from another protein largely rescued the phenotypes identified in the mutant including deficiencies in the spindle checkpoint and mitotic timing (22). This mutant retains the capacity to not only interact with free tubulin but also had reduced phase separation to levels similar to the CEN-Borealin^LLPS^ (Fig. S5). Importantly, no MT bundles were detected from condensates of the CEN^MTBD^ (Fig. 5*B*). These data suggest that the bundles of MTs that emerge from condensates require the MT-binding activity on the N terminus of Borealin.

To investigate the role of Borealin phase separation and MT binding in formation of MT bundles independently of MT nucleation, we incubated CEN-Borealin^wt^ and mutants with GMpCpp-stabilized MTs and quantified the width of MT bundles. CEN-Borealin^wt^ generated on average bundles of 0.46 μm width, whereas bundles generated by mutants were significantly thinner (0.35 μm and 0.36 μm width for Borealin^LLPS^ and MTBM mutant, respectively) and similar to the measured width of single GMpCpp in this assay, which is 0.34 μm (Fig. 5*C*). Both mutants similarly sequestered αβ-tubulin dimers in the absence of GTP (Fig. S5*D*) consistent with the findings that activities in the CPC beyond simple tubulin concentration in condensates are required for bundle formation. These results suggest that both the MT binding and LLPS activities of the Borealin subunit of the CPC contribute to the formation of MT bundles *in vitro*. We note that our mutants are not able to cleanly separate the two activities, but the simplest interpretation from these data and our published work is that the Borealin^LLPS^ mutant is deficient at phase separation and the Borealin^MTBM^ is deficient at both phase separation and MT binding.

### Borealin-dependent liquid–liquid phase separation and MT binding regulates midzone MTs

**Figure 6:**
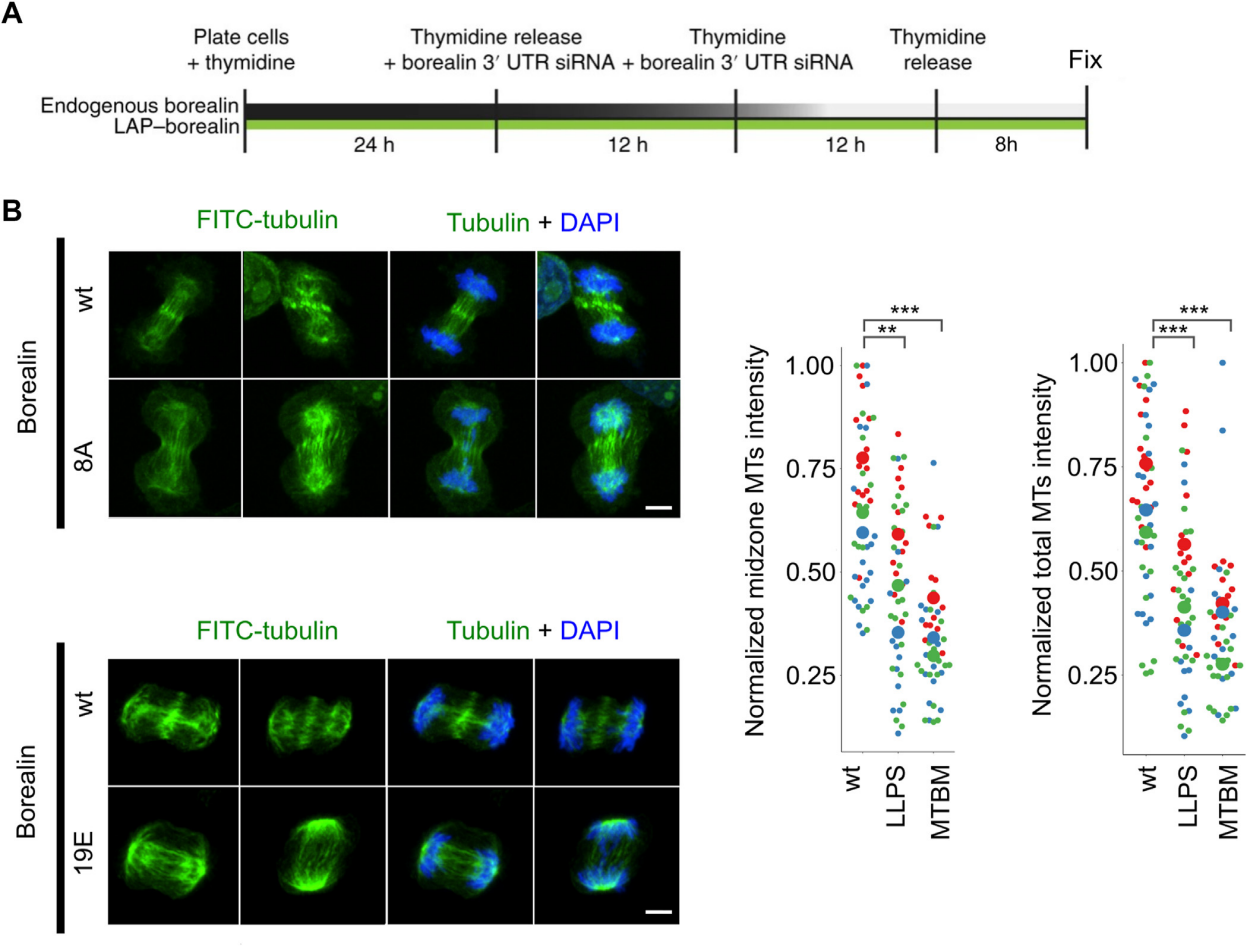
**CPC phase separation and MT bundling are important for formation of bundled structures on the anaphase spindle.***A*, experimental design to visualize spindles in the first mitosis after knockdown and replacement of Borealin by siRNA in HeLa cells. *B*, fluorescence images of HeLa cells with endogenous Borealin replaced with WT or indicated Borealin mutants and stained for α-tubulin and DAPI. Maximum intensity projections of representative images are shown; scale bar represents 5 μm. Experiment was repeated three times, and the different colors in the quantifications represent experimental replicates; data were normalized between experimental replicates by dividing the mean values of intensity of signal from MTs by the maximum of the total intensity within each experimental replicate; CEN-Borealin^wt^ (n = 28, 38, 32); CEN-Borealin^LLPS^ (n = 26, 28, 42); and CEN-Borealin^MTBM^ (n = 26, 30, 38). *p*^wt-LLPS^ = (0.015, 0.00022, 0.0058); *p*^MTBM^ = (1.77× 10^−6^, 6.9 × 10^−5^, 3.3 × 10^−8^). For statistical analysis, Welch's *t* test with Bonferroni correction was applied; ∗*p* < 0.05, ∗∗*p* < 0.01, ∗∗∗*p* < 0.001. CPC, chromosomal passenger complex; DAPI, 4′,6-diamidino-2-phenylindole; MT, microtubule.

We employed the phase separation and MT-binding mutants to test the importance of the CPC’s MT-bundling activities in HeLa cells. Previously characterized cell lines stably expressing similar levels of siRNA-resistant Borealin WT-GFP or either of the mutant proteins were arrested in S-phase and depleted of endogenous Borealin by siRNA (22, 24). The cells were released from the block, fixed in glutaraldehyde to preserve MT structure, and analyzed during the first mitotic transition (Figs. 6*A* and S6*A*). Previous characterization of these mutants has shown that the localization of the CPC is reduced by these mutations at inner centromeres and midzones (24, 28). The overall spindle structure appeared to be unaffected; however, the midzones of cells mutated for MT binding or phase separation contained approximately twofold less tubulin than those of cells rescued with Borealin^wt^ (Fig. 6*B*). The amount of tubulin in the central region between segregating chromatin masses was quantified relative to total tubulin in the anaphase spindles, which includes midzone MTs as well as bundles of MT at the cortex (3).

These changes in the midzone and cortical MT populations are unlikely to be an indirect effect of changes to Aurora B activity since adding an Aurora B inhibitor (AZD1152-HQPA-Barasertib) did not affect the amount of midzone MTs (Fig. S6*C*). Together, these data suggest that the MT bundles at the anaphase midzone and cortex are dependent on the CPC and require regions on Borealin required for MT binding and phase separation. They also suggest that this is independent of Aurora kinase B activity, although this requires further study.

## Discussion

**Figure 7:**
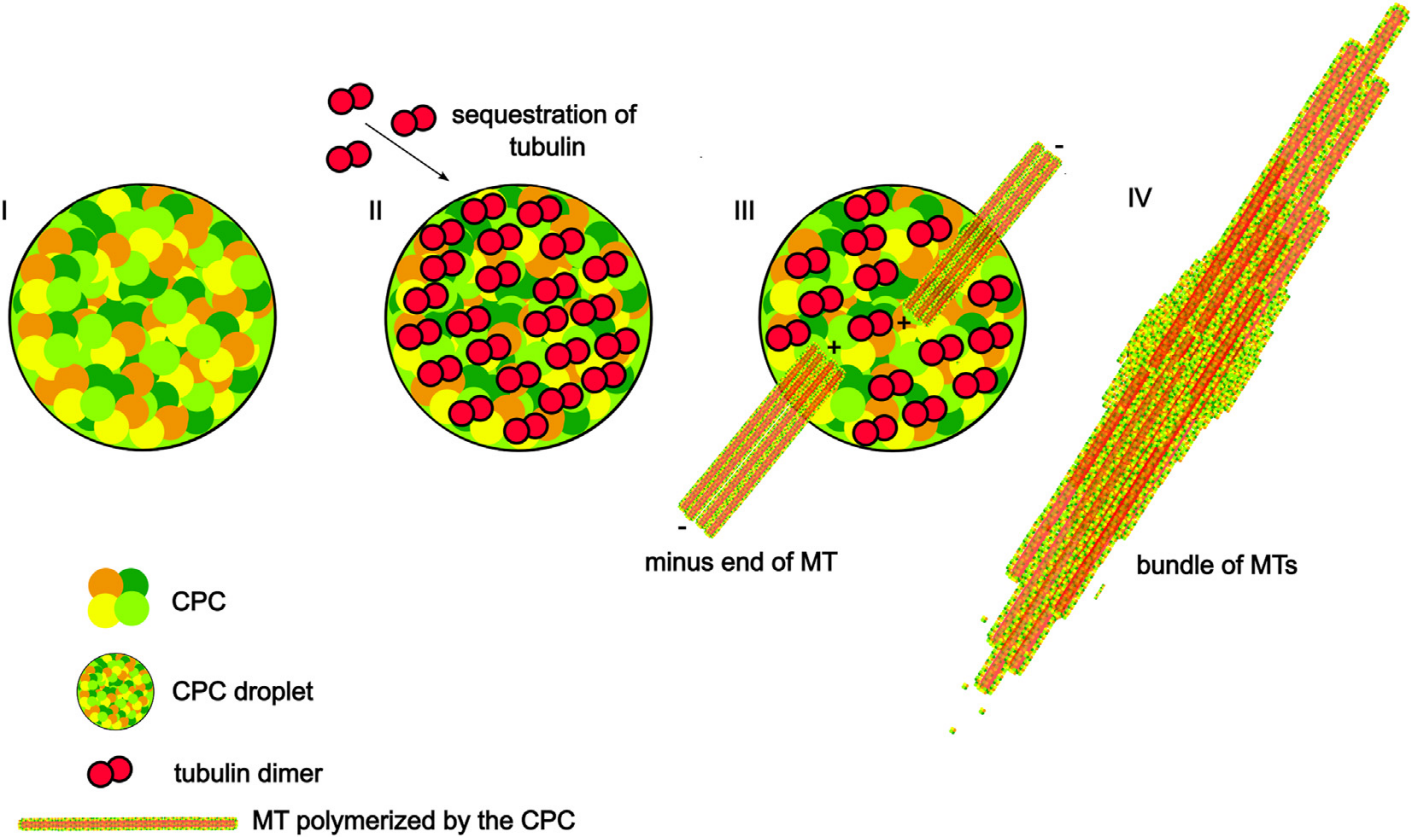
**A model of LLPS of the CPC leads to formation of bundle of MT.** The CPC in liquid–liquid demixed state (I) sequesters free tubulin (II) increasing the tubulin concentration. That allows for nucleation and MT polymerization in such a way that MTs are parallel with minus end pointing outward (III). Such process leads to formation of MT bundles (IV). CPC, chromosomal passenger complex; LLPS, liquid–liquid phase separation; MT, microtubule.

We demonstrated that the CPC has a surprising *in vitro* activity to generate parallel bundled MTs *in vitro*. Our data suggest that concentrated CPC employs its ability to bind MTs to organize parallel bundled MT structures that are central features of the anaphase midzone. A simple *in vitro* system of LLD-CPC, tubulin, and GTP generated robust tapered parallel bundles of MTs that elongated from condensates over time (Fig. 7). Both CPC phase separation driven by Borealin and MT binding by Borealin are important to generate the structures *in vitro* and for maintaining proper density of MTs in the midzone during anaphase. In broader context, our results extend the emerging concept that protein LLPS provides a mechanism to concentrate molecules to regulate cellular process and to act as organizational hubs for formation of ordered cellular structures (33).

In our experimental setup, MT nucleation and bundling were dependent on CPC condensates and specific interactions between the CPC and tubulin. Indeed, there are two documented MT-binding regions within CPC: one on the N-terminal fragment of Borealin (22) and another within the INCENP SAH domain (23). Using a CEN domain construct with MT-binding deficient Borealin, we demonstrated that interactions between tubulin and Borealin are important to initiate MT nucleation and bundling. Tubulin dimers assemble into longitudinal oligomers when they are above a critical concentration that subsequently form a sheet through lateral association with tubulin dimers (34). We suggest that the CPC condensates concentrate tubulin above its critical concentration to generate MTs, but we also show that the bundling activity is more complex using mutants that concentrate tubulin but not bind MTs. We suggest that the CPC stabilizes the generated MTs through its bundling activity. Another possibility is that by phase separation CPC creates a space so dense in tubulin-binding motifs that tubulin dimers interact with each other to form MT seeds, and to begin nucleation *in vitro*. We have no evidence that the CPC is sufficient to nucleate MTs *in vivo*, and we currently favor models where the CPC works with MT nucleators such as γ-TuRC in cells. We noticed that the protofilament numbers of MTs generated by the CPC *in vitro* are different from those generated by the γ-TuRC complex *in vivo*, which is in agreement with a major role of γ-TuRC in MT nucleation process. An important area of future research will be to test if the γ-TuRC also works in combination with the CPC (35).

We previously demonstrated that the LLD activity of CPC is important for its mitotic functions such as correcting kinetochore–MT attachments and maintaining the spindle checkpoint (24). In this work, we show that the CPC combines phase separation and its ability to interact with tubulin to form parallel MT bundles. Our *in cell* analysis demonstrates that mutants deficient for both Borealin-dependent phase separation and MT binding both reduce formation of midzones in HeLa cells, which are structures that the CPC localizes to and are formed by bridging two parallel bundles of MTs. We note that both these mutants have been previously shown to decrease the amount of CPC at midzones (24, 28), which complicates interpretation. It has been suggested that midzones might “push” on the back of segregating chromatids as an additional force during anaphase (36, 37). Since single MTs bend, it is difficult to imagine how single MTs could generate a pushing force. In contrast, bundles of MTs could work together to generate a pushing force. It will be important to test if CPC mutants deficient in bundle formation are deficient at segregating chromatids.

Surprisingly, our analysis suggests that bundles grow from condensates with their minus ends extruding, which represents growth opposite of standard MTOCs including centrosomes and Golgi. How cells employ this new enzymatic activity is still unclear. We note that in monopolar spindles and after nocodazole washout, kinetochores generate tapered MT bundles known as “preformed kinetochore fibers” that are MT bundles that have been suggested to emerge from kinetochores with their minus ends extruding outward (38). We have previously shown that the CPC interacts with preformed K-fibers more than other spindle MTs (19). It will be important to test if the CPC enzymatic activities identified here underlie the formation of preformed K-fibers in the future.

In summary, we have discovered that the CPC extends tapered MT bundles from a central organizing condensate *in vitro*, and analysis of mutants deficient in this activity in cells suggests that this activity contributes to midzone formation. These data also demonstrate a mechanism for an MTOC to generate MTs with the opposite polarity of centrosomes and traditional MTOCs. How the CPC is able to allow plus-end growth within a condensate while “pushing” the bundles outward will be an important area of future research.

## Experimental procedures

### Protein purification

Chromosomal passenger complex: *Xenopus* INCENP and Aurora B (amino acids 33–368) genes were cloned into pMCSG11 vector as a bicistronic construct with an N-terminal 6xHIS affinity tag on the INCENP protein. *Xenopus* Survivin-B and Borealin/Dasra-A were cloned into pET15b as a separate bicistronic vector. In the case of CPC-GFP construct, the GFP tag was on the N terminus of Survivin. The recombinant CPC or CPC-GFP were overexpressed in *E. coli* strain BL21-CodonPlus (DE3) Magic containing both vectors in the presence of carbenicillin (100 μg/ml), kanamycin (50 μg/ml), and chloramphenicol (34 μg/ml). Bacteria were induced at absorbance of 0.6 with 0.15 mM isopropyl-β-d-thiogalactoside followed by adding 80 μM ZnCl_2_ per 1 l of LB medium. Cells were incubated with shaking for 16 h at 18 °C. Cells were harvested, and pellets were suspended in lysis buffer (2 mM imidazole, 50 mM Tris, pH 7.9, 500 mM NaCl, 5% glycerol, and 0.5 mM Tris(2-carboxyethyl)phosphine (TCEP)) containing cOmplete Protease Inhibitor Cocktail (Roche), lysozyme, and DNAse. Cells were lysed using an EmulsiFlex-C3 homogenizer. Lysates were clarified by ultracentrifugation at 30,000 rpm for 40 min at 4 °C. The supernatant was incubated with Ni^2+^ resin (Qiagen) at 4 °C. Beads were washed in a buffer containing 10 mM imidazole, 50 mM Tris, pH 7.9, 500 mM NaCl, 5% glycerol, 0.5 mM TCEP, and proteins were eluted with a buffer composed of 250 mM imidazole, 50 mM Tris, pH 7.9, 500 mM NaCl, 5% glycerol, and 0.5 mM TCEP. Protein was separated on Superdex 200 in a buffer containing 50 mM Tris, pH 7.9, 500 mM NaCl, 5% glycerol, and 0.5 mM TCEP. Fractions containing CPC were pooled and concentrated in a centrifugal filter unit with cutoff of 30 kDa (Amicon), aliquoted, and stored at −80 °C.

#### Kinesin-1-GFP

Plasmid coding for kinesin-1-GFP was purchased from Addgene repository (#129761). The protein was expressed in BL21(DE3)RIL cells. Bacteria were induced at an absorbance of 0.6 with 0.15 mM isopropyl-β-d-thiogalactoside in LB medium. Cells were incubated with shaking for 16 h at 18 °C. Cells were harvested, and pellets were suspended in a lysis buffer (2 mM imidazole, 50 mM Tris, pH 7.9, 200 mM NaCl, 1% glycerol, 0.5 mM TCEP, and 4 mM MgSO_4_) containing cOmplete Inhibitor Protease Cocktail, lysozyme, and DNAse. Cells were lysed using an EmulsiFlex-C3 homogenizer. Lysates were clarified by ultracentrifugation at 35,000 rpm for 40 min at 4 °C. The supernatant was incubated with Ni^2+^ resin at 4 °C. Beads were washed in a wash buffer containing 10 mM imidazole, 50 mM Tris, pH 7.9, 500 mM NaCl, 1% glycerol, 0.5 mM TCEP, and 4 mM MgSO_4_, then washed again with a wash buffer supplemented with 50 μM ATP. Protein was eluted with a buffer composed of 80 mM Pipes (pH = 7.0), 4 mM MgSO_4_, 300 mM imidazole, and 50 μM ATP. Protein was separated on Superdex 200 in a buffer containing 80 mM Pipes (pH = 7.0), 4 mM MgSO_4_, and 1 mM EGTA. Fractions containing kinesin-1-GFP were pooled and concentrated.

The CEN complex is composed of human INCENP (1–58 amino acids), full-length human Survivin, and full-length human Borealin. CEN-Borealin^wt^, CEN-Borealin^LLPS^, and CEN-Borealin^MTBM^ were purified using the same protocol as for purification of the CPC. The CEN protein was expressed from pET28b vector in BL21(DE3) Rosetta cells.

### Phase separation assay

CPC phase separation was induced by incubating 1 μM CPC in a buffer containing 150 mM NaCl and 20 mM Hepes (pH = 7.2), or a buffer composed of 500 mM NaCl and 20 mM Hepes (pH = 7.2) supplemented with 7% PEG3350 to serve as a crowding agent (Fig. 1*C*). For consistency, in all other experiments, CPC phase separation was induced by mixing with a buffer composed of BRB40 (40 mM Pipes, 0.5 mM MgCl_2_, 0.5 mM EGTA, pH 6.8), 1 mM GTP, 1 mM DTT, and 7% PEG3350. For time-lapse imaging of CPC droplet fusion, CPC phase separation was induced with a BRB40 mix containing 7% PEG3350, the mixture was transferred to microscopic chambers, and droplet fusion was immediately imaged under ×63 objective (Plan Apo) DIC on a ZEISS AxioObserver Z1 microscope.

To compare the phase separation capacity of CEN-Borealin^LLPS^ and CEN-Borealin^wt^, phase separation of each protein was induced by diluting CEN into buffer containing 150 mM NaCl. A sample from this mixture was immediately transferred into a coverslip flow chamber made of small channels separated by paraffin wax. DIC images of condensates were acquired after a 15 min incubation of this chamber at room temperature, which allowed condensates to settle to a common Z plane as well as to relax and fuse.

### MT polymerization assay

About 1 μM CPC in size-exclusion chromatography buffer was mixed with BRB40 buffer (40 mM Pipes, 1 mM MgCl_2_, 1 mM EGTA, pH  6.8 with NaOH), 1 mM GTP, 1 mM DTT, and 7% PEG3350 to induce phase separation. Then 2 μM of α/β tubulin dimers (PurSolutions) in BRB80/DTT buffer was added, and the reaction was kept at room temperature for 40 min. Unlabeled tubulin was mixed with labeled tubulin at 10:1 ratio. Rhodamine-labeled tubulin was purchased from Cytoskeleton, and Cy-5-labeled tubulin was purchased from PurSolutions. Soluble tubulin was added to the mixture immediately after induction of CPC phase separation. Polymerized MTs were transferred onto cover glasses or chambers (Grace Bio-Labs) and observed using a 63× objective on a Zeiss Observer Z1 wide-field microscope by fluorescence and DIC imaging or using a 100× objective on a LEICA Thunder/TIRF. To prevent shearing forces during transfer of polymerized MTs from a tube to the cover slip for imaging tips that were cut by a sharp razor, 2 mm from the top of a tip were used.

MT polymerization by CEN complex was assayed by mixing CEN^wt^, CEN^LLPS^, and CEN^MTBM^ at 7 μM concentration with BRB40, 1 mM GTP, 1 mM DTT, 7% PEG3350, and tubulin for 40 min at room temperature. The reaction was transferred between two cover glasses and imaged on 63× objective on a ZEISS AxioObserver Z1 microscope.

### Kinesin-1 motility assay—motor-PAINT assay

Cy5-labeled MTs were polymerized in the presence of GTP and phase-separated CPC. Cy-5 tubulin was from PurSolutions. Reaction mixture was transferred into coverslip-bottomed CultureWell (Grace BioLabs), and then solution of glucose/oxidase system (40 mM glucose, 130 mg/ml glucose oxidase, and 24 mg/ml catalase), 1 mM ATP, and 30 nM of kinesin-1-GFP was added. Kinesin movements were imaged using 100× objective in a TIRF mode with excitation at 480 nm on the LEICA Thunder microscope. Images were taken every 220 ms. Analysis was done in FIJI (National Institutes of Health and the Laboratory for Optical and Computational Instrumentation (LOCI, University of Wisconsin)).

### Partition coefficient of tubulin and peptides into CPC coacervates

Partition coefficient for H3T3ph(1–21)-FITC, H3T3(1–21)-FITC, and rhodamine tubulin into CPC condensates was measured according to a published protocol (21). About 400 nM of peptide, 400 nM of enhanced GFP, or 2 μM of α/β tubulin dimers (1:10 rhodamine labeled to unlabeled) in BRB80/DTT were added to CPC condensates that were generated by mixing 1 μM of the CPC with BRB40 buffer, 1 mM DTT, and 7% PEG3350. Condensates were imaged on 63× objective on a ZEISS microscope. Fluorescence signals were calculated in FIJI. Partition coefficients were calculated by dividing the fluorescence signal per unit area inside the coacervates by the fluorescence signal per unit area outside the coacervates after subtracting the background fluorescence. Background fluorescence was calculated by imaging the coacervates in the absence of fluorescent agent molecules.

### Bundling of MTs by CEN subcomplex

GMpCpp MTs were prepared by incubating 200 μM α/β tubulin dimers with 1 mM of GMpCpp for 1 h at 37 °C in the BRB80/DTT buffer. MTs were pelleted for 5 min at 27 °C using Sorvall centrifuge and S100-AT3 fixed angle rotor. The pelleted MTs were resuspended in BRB80/DTT buffer. About 100 nM of GMpCpp-stabilized and rhodamine-labeled MTs were mixed with 4 μM CEN^wt^, CEN^LLPS^, and CEN^MTBM^, which is below critical concentration for CEN. After 2 min incubation, reactions were transferred in between cover slides and imaged on the 100× objective using TIRF mode on LEICA Thunder microscope. Bundling reactions were performed in BRB80/DTT buffer. The width of MT bundles was measured in FIJI. The selected MT bundle was traced by segmented line and straightened using built-in function in FIJI. MT bundle was aligned vertically, background was subtracted, and a two-dimensional graph of the intensities of pixels along a line within the bundle was generated using the plot profile function. Gaussian profile was fitted, and the parameter describing the full width at half maximum of a peak was used as a measure of the width of a bundle.

### Kinase assay and immunoblotting

About 1 μM of recombinant CPC was mixed with 2 μg of glutathione-*S*-transferase-histone H3 (1–21 amino acids) fragment, 10 mM ATP, 10 mM MgSO_4_, 2 mM DTT, and 20 mM Hepes (pH = 7.2), and 7% PEG3350 and incubated at 37 °C for 5 min. The reaction mixture was resolved on 15% SDS-PAGE gel and blotted with antibodies against histone H3 S10ph (Cell Signaling Technologies; 1:1000 dilution).

### EM data collection and MT reconstruction

MTs generated by the CPC (∼3.0 μl) were applied to discharged lacey carbon grids and plunge frozen in liquid propane. Frozen grids were imaged in a Titan Krios (ThermoFisher) at 300 keV with a K3 camera (Gatan, 2.6 Å per pixel). MT reconstruction was done in FIJI using a TubuleJ plugin accordingly (39, 40). The 3D reconstruction was visualized with UCSF Chimera (41), and the obtained volume corresponding to MTs was compared with the tubulin model (Protein Data Bank ID: 1TUB). The structural features including the H1-S2-loop pointing inward and the C-terminal α-helices pointing outward, and densities corresponding to the lateral interactions, were taken into consideration while assessing the agreement between the volume from 3D reconstruction and the overall shape of the 1TUB model.

### CPC mass spectrometry analysis

xCPC protein sample (composed of proteins: INCENP-UNP: O13024; AuroraB UNP: Q6DE08; Dasra/Borealin UNP: Q4V7H8; and Survivin UNP: Q8JGN5) was prepared as two 5 μg aliquots for trypsin and GluC peptide mapping as previously described (37). Samples were analyzed as single injections by nanoLC–MS/MS (nanoRSLC; ThermoFisher) coupled to an Orbitrap Eclipse using a 120 min reversed phase gradient with resolution settings of 120,000 and 15,000 (at an *m/z* 200) for MS1 and MS2 scans, respectively. Selected peptides were fragmented using stepped high energy collisional dissociation (20, 30, and 40%). Tandem mass spectra were analyzed according to a label-free quantitation proteomic strategy using quantitative Proteome Discoverer (version 2.5.0.400; ThermoFisher) with the Byonic (version 4.1.10; Protein Metrics) and Minora nodes and using a database consisting of *E. coli* and *Xenopus* CPC protein sequences to objectively identify all CPC and background *E. coli* proteins present in the purified sample (42, 43). Mass tolerances of 10 and 20 ppm were used for matching parent and fragment masses, respectively. Spectra were searched with a fixed modification of carbamidomethyl (C) and variable modifications of phosphorylation (S, T, Y). Protein label-free quantitation abundances were calculated with the Precursor Ions Quantifier node in Proteome Discoverer (ThermoFisher Scientific) using Summed Abundances as the method of Protein Abundance Calculation, where protein abundances are calculated by summing the abundances of the connected peptide groups. Protein abundance normalization was not performed. Tandem mass spectra were then analyzed according to a peptide mapping strategy in which MS/MS spectra were extracted and charge state deconvoluted, then searched with a database consisting of the top 20 Byonic-identified proteins (sorted by log probability and accounting for greater than four orders of magnitude of the highest abundance proteins detected by Proteome Discoverer) using the MassAnalyzer algorithm embedded in BioPharma Finder software (version 4.1.53.14; ThermoFisher) (43). Searches were performed with an MS noise level of 2000 and an S/N threshold of 10, parent ion tolerance of 10 ppm, and an MS/MS minimum confidence level of 80%. Trypsin was selected as the protease with a “high” specificity setting. Variable modifications of carbamidomethyl (C), oxidation (M), deamidation (N, Q), and phosphorylation (S, T, Y) were specified. Phosphopeptide identifications were manually validated. Apart from the four proteins from the CPC, other *E. coli* proteins were present in the sample (Table S1).

### Analysis of CPC subunits in condensates

CEN and CPC protein samples were diluted into buffer containing 150 mM NaCl in test tubes to induce phase separation, followed by centrifugation at 21,130 RCF for 5 min in a temperature-controlled benchtop centrifuge at 23 °C. Supernatants were removed and discarded, and the pelleted material was resuspended by boiling for 10 min in Laemmli sample buffer before separation by SDS-PAGE. Input samples were prepared by diluting an equal protein mass directly into sample buffer.

### CPC dephosphorylation reaction

About 10 μmol purified CPC were incubated with 2.5 μmol λ protein phosphatase (New England Biolabs) in PMP buffer (50 mM Hepes, 2 mM DTT, 0.01% Brij 35; New England Biolabs) supplemented to 500 mM NaCl to promote solubility of the CPC and in the presence of 1 mM MnCl_2_. Reactions were stopped by addition of Laemmli sample buffer, after which samples were boiled for 10 min and separated by SDS-PAGE.

### Cell culture

HeLa T-REx cells (ThermoFisher Scientific) stably expressing LAP-Borealin^wt^, LAP-Borealin^LLPS^, and LAP-Borealin^MTBM^ (19) were grown in Dulbecco’s modified Eagle’s medium (DMEM; Invitrogen) supplemented with 10% fetal bovine serum (FBS; Gibco) in the presence of 5% CO_2_ in a humidified incubator at 37  °C.

### siRNA transfection

For knockdown and replacement experiments, Borealin 3′ UTR siRNA (AGGUAGAGCUGUCUGUUCAdTdT) was transfected using RNAiMAX (Invitrogen) according to the manufacturer’s protocol. Mitotic phenotypes were analyzed in the first mitosis after complementation. Borealin stable cell lines were plated in the presence of 2 mM thymidine for 24 h. Next, the fresh DMEM/10% FBS media were added, and cells were transfected with siRNA and incubated for 12 h. Next, media were replaced, and cells were incubated with DMEM/10% FBS with 2 mM thymidine, transfected with siRNA, and incubated for 12 h. Next, cells were released from thymidine in fresh media DMEM/10% FBS for 9 h and fixed. For Aurora B inhibition experiments, 1 μM AZD1152-HQPA (Barasertib; SelleckChem) was added for 30 min before fixation in glutaraldehyde after cells were treated with siRNA following the protocol described previously.

### Immunofluorescence

Cells were plated on cover slides coated with poly-l-lysine (Sigma). For midzone staining and analysis, cells were extracted for 30 s with 0.5% Triton X-100 in PHEM buffer (25 mM Hepes, 60 mM Pipes, 10 mM EGTA, and 4 mM MgCl_2_, pH  6.9) and then fixed in 2.5% glutaraldehyde in PHEM buffer for 10 min. Reaction was quenched with sodium borohydride for 7 min. Slides were washed three times with Tris-buffered saline with Tween-20 (TBS(T)) buffer, blocked for 1 h in 3% BSA/TBS(T) buffer, incubated with antitubulin DM1α-FITC-labeled antibody for 1 h. Slides were washed four times with TBS(T) buffer, the last wash contained 4′,6-diamidino-2-phenylindole (0.5 μg/ml). Next cells were washed twice with water and mounted with ProLong Gold Antifade (ThermoFisher Scientific).

### Image analysis

Images were acquired with a 63×/1.4 numerical aperture objective on a Zeiss Observer Z1 wide-field microscope. Image analysis was done in FIJI. The Z-stacks were projected using maximum intensity algorithm. Confocal images were acquired with a 60×/1.4 numerical aperture objective on a Nikon ECLIPSE Ti2-E and processed in NIS-Elements software (Nikon). Deconvolution and 3D projections of representative images were generated by manipulation in NIS-Elements.

#### Midzone analysis

To analyze the level of MTs in midzone of anaphase cells, the mean values of intensity of signal from MTs (the region between separating chromatids or intensity of the whole cell) labeled with FITC were divided by the maximum of the total intensity within each experimental replicate.

#### motor-PAINT assay

Kinesin-1 movements along polymerized MTs were analyzed in FIJI using multikymograph plugin. The polymerized MTs were traced with line drawn in a direction to the droplet producing a kymograph from which the direction of the kinesin was read. If the kinesin signal was changing to the right of the image that was considered as a movement toward the droplet.

### Statistics and reproducibility

All the key experiments were repeated multiple times as indicated in the figure legends. Experiments for Figures 1, *E* and *F*, 2, *B* and *D*, 3, 4, and 5*C* were performed twice independently with similar results. Experiments for Figures 5*B* and 6*B* were performed three times independently with similar results. Indicated statistical analysis was performed using R (version 3.6.3). Experiments in Figures 1, *C* and *D*, 2, *A*, *C*, and *D*, S2*A*, 4, and S4*B* were done with the CPC construct only; experiments in Figures 2*B* and 3 were done with GFP-CPC mixed with CPC at 1:500 ratio; there are no experiments in the article where only CPC-GFP protein was used.

## Data availability

All raw data will be made available upon reasonable request. Source data are provided with this article.

## Supporting information

This article contains supporting information.

## Conflict of interest

The authors declare that they have no conflicts of interest with the contents of this article.
