# Supplementary material for: Chromosomal passenger complex condensates generate parallel microtubule bundles in vitro
Source: J Biol Chem. 2024 Jan 23;300(3):105669. doi: 10.1016/j.jbc.2024.105669 (PMC10876603; doi:10.1016/j.jbc.2024.105669)
Supplement: Supporting Figure and Table [file mmc1.docx]

**Supplementary materials**

**Chromosomal passenger complex condensates generate parallel microtubule bundles in vitro**

Ewa Niedzialkowska^1^, Tan M. Truong^2^, Luke A. Eldredge^2^, Aamir Ali^2^, Stefanie Redemann^1,3,4^, P. Todd Stukenberg^1*^

^1^Department of Biochemistry and Molecular Biology, University of Virginia, School of Medicine, Charlottesville, VA, USA

^2^Department of Cell Biology, University of Virginia, School of Medicine, Charlottesville, VA, USA

^3^Department of Molecular Physiology and Biological Physics, University of Virginia, School of Medicine, Charlottesville, VA, USA

^4^Center for Membrane and Cell Physiology, University of Virginia, School of Medicine, Charlottesville, VA, USA

*corresponding author

**Inventory of supplementary materials:**

**Supplementary 1.** Biochemical characterization of recombinant CPC prep.

**Supplementary 2.** MTs are polymerized and bundled *in vitro* by the phase-separated CPC.

**Supplementary 4.** Kinesin-1-GFP purity and motorPAINT analysis.

**Supplementary 5**. Purity of recombinant CEN-Borealin^wt^, CEN-Borealin^8A^, CEN-Borealin^R17E,R19E,R20E^ protein preps.

**Supplementary 6.** Western blot of Borealin siRNA knock-down and replacement and immunofluorescence analysis of Borealin depleted cells.

**Supplementary data #7.** Uncropped versions of gels and blot presented in this study.

**Supplementary data #8.** Amino acid sequences of proteins used in the study

**Supplementary data #9.** Table with non-CPC proteins detected by MS analysis

**Supplementary Figure 1**

**
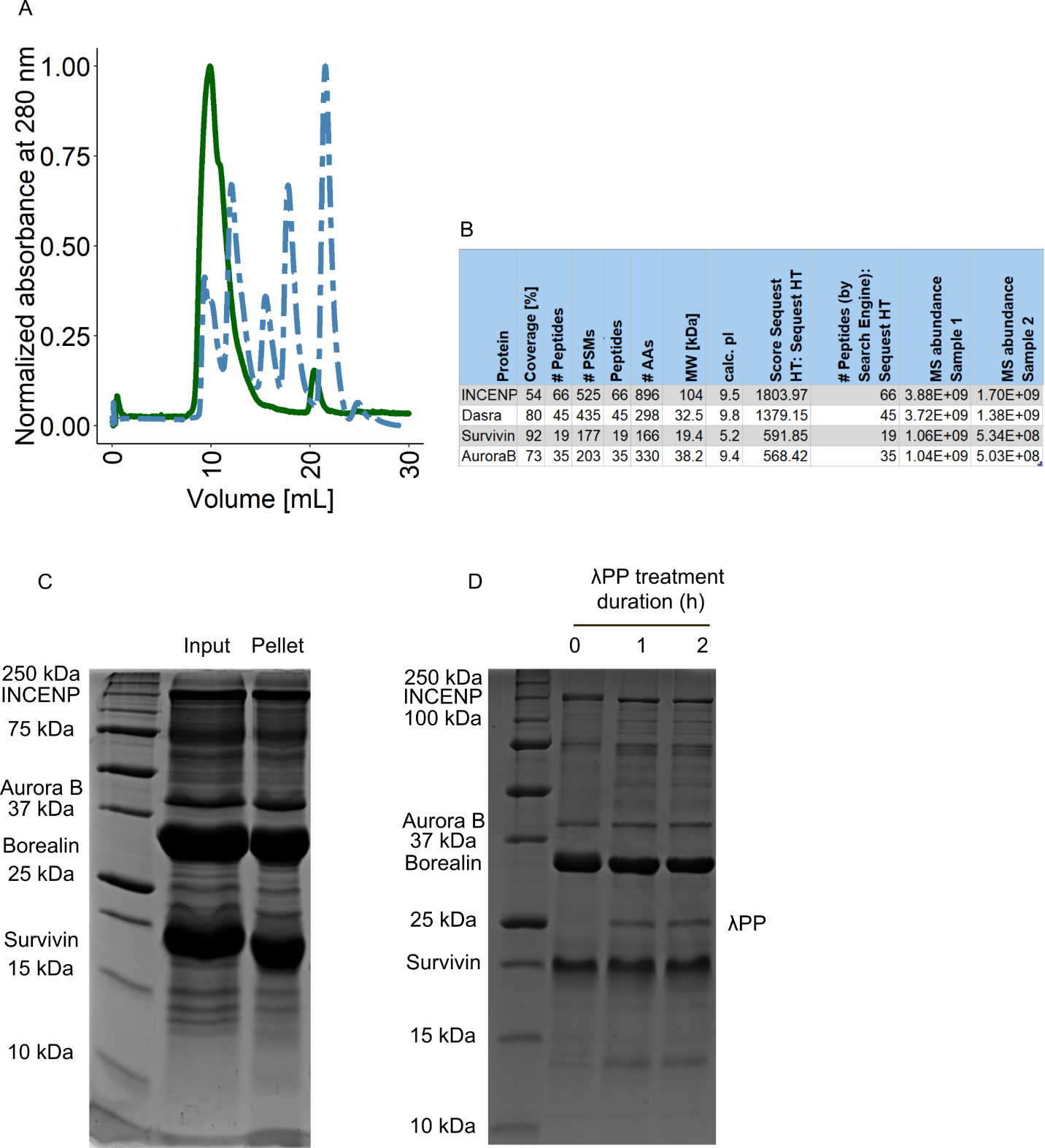
**

**Supplementary 1. Biochemical characterization of recombinant CPC prep.** A. Size exclusion profile of the CPC. Protein complex was separated on Superdex 200 10/300 GL attached to BioLogic DuoFlow™ Medium-Pressure Chromatography System (Bio-Rad) in a buffer composed of 20 mM HEPES 7.2, 500 mM NaCl, 2% glycerol, 0.5 mM TCEP. CPC elution profile is marked as solid line; elution profile of protein standards is marked as dotted line. Standards are 670, 158, 44, 17 kD molecular weight. B. Mass spectrometry results of the CPC protein prep. C. To verify that all components of the purified CPC complex are retained in condensates, phase separate was induced by dilution into 500 mM NaCl + 7% PEG-3350. Condensed material was isolated by centrifugation, separated by SDS-PAGE, and stained with Coomassie (“Pellet” lanes). An equal mass of soluble protein was added directly to sample buffer (“Input” lanes) for comparison. D. Purified CPC was incubated with λ protein phosphatase for the indicated amounts of time, and the apparent molecular weights of the components tracked by SDS-PAGE. Note that INCENP and Borealin are both initially visible as doublet bands which collapse upon phosphatase treatment.

Note about stoichiometry of the CPC prep. Purification of the CPC has been extremely challenging. The stoichiometry of the prep is not ideal so we include gels (supplementary figure 1C and D) where we have vastly overloaded the proteins on a gel so that the reader can evaluate the preps. When we quantified the stoichiometry by mass spec there were similar amounts of INCENP and Borealin and about 2-fold less Survivin and Aurora B (Supplemental figure 1B sample 2). The 6his tag is on the N-terminus of INCENP which binds Borealin and Survivin. In contrast Aurora B binds the C-terminus of INCENP and thus the amount of Aurora B reflects the amount of near full-length INCENP. By gel (where we are looking at full-length proteins) INCENP is lower stoichiometry and there are many lower bands. We therefore conclude that the proteins are close to stoichiometric but there is likely to be some degradation of the INCENP protein leading to the gel appearance. We are confident that the stoichiometry differences are not causative of the ability to generate microtubule structure since we have seen the in vitro activity in multiple preps including the ISB subfragment that is stoichiometric as shown in figure 5.

**Supplementary Figure 2**


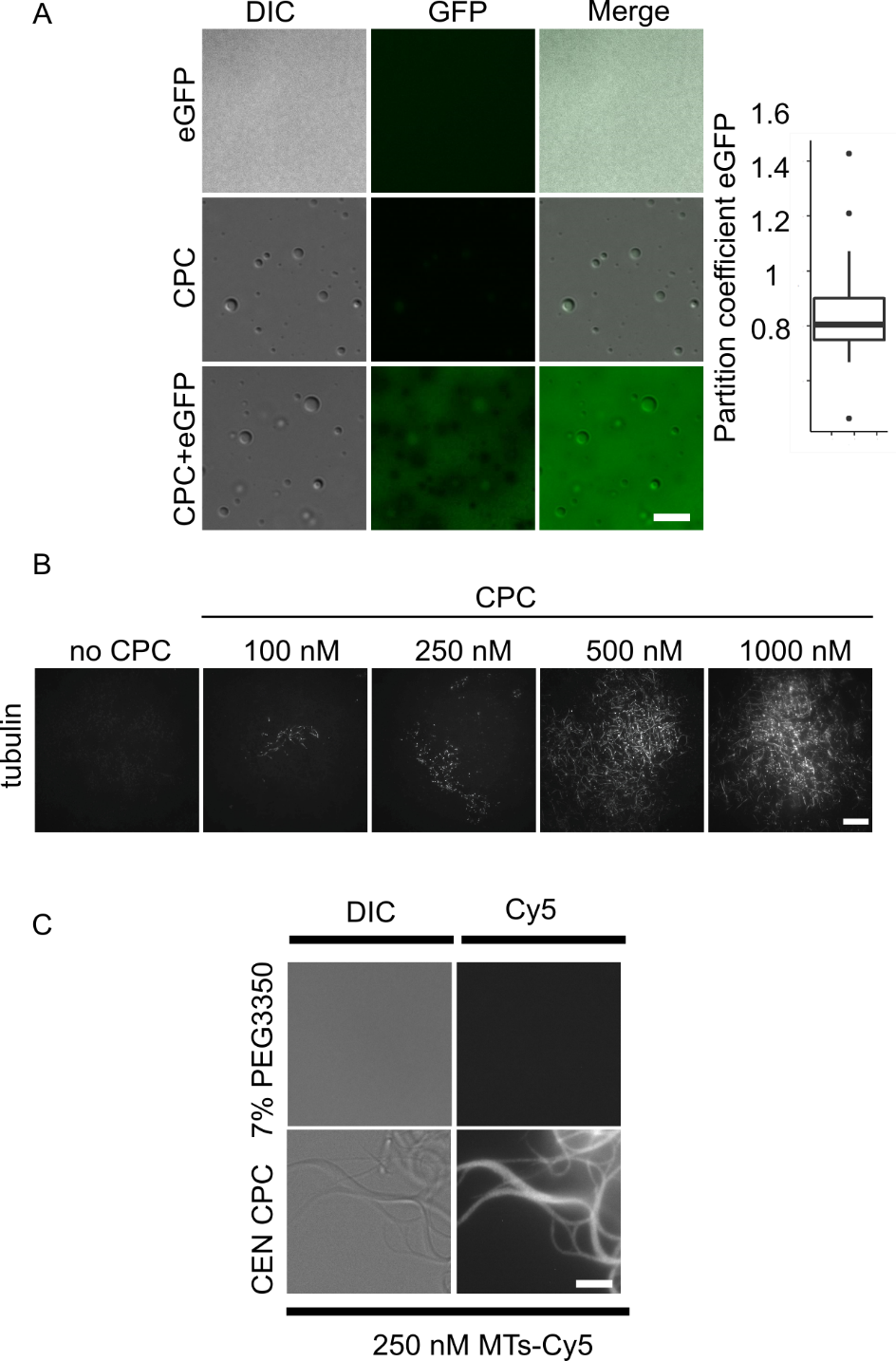


**Supplementary Figure 2. MTs are polymerized and bundled *in vitro* by the phase-separated CPC.** A. GFP is not enriched in CPC condensates, which is a control for the enrichment of tubulin in Figure 2A. DIC and fluorescence images showing partitioning of GFP into CPC condensates; (n=69); objective 63x; scale bar = 5µm. Box and whisker graph represents the median (central line), 25th-75th percentile (bounds of the box), and 5th-95th percentile (whiskers). B. The density of MT structures generated when the CPC is incubated with tubulin and GTP is dependent upon the concentration of CPC. Rhodamine labelled α/β-tubulin dimers were incubated with various concentrations of LLPS CPC, and resulting MT bundles were imaged by TIRF microscopy; objective 100x; scale bar 20 µm. Images present the largest observed structure from each concentration. Experiment was repeated twice and for each repetition 4 to 8 fields of view were imaged. C. Bundling of polymerized MTs depends upon CPC, but not PEG. DIC and fluorescence images of GMpCpp stabilized MTs labelled with Cy5 incubated with 7% PEG3350 or 10 µM CEN subcomplex in the presence of 7% PEG3350; objective 63x; scale bar = 5µm.

**Supplementary Figure 4**


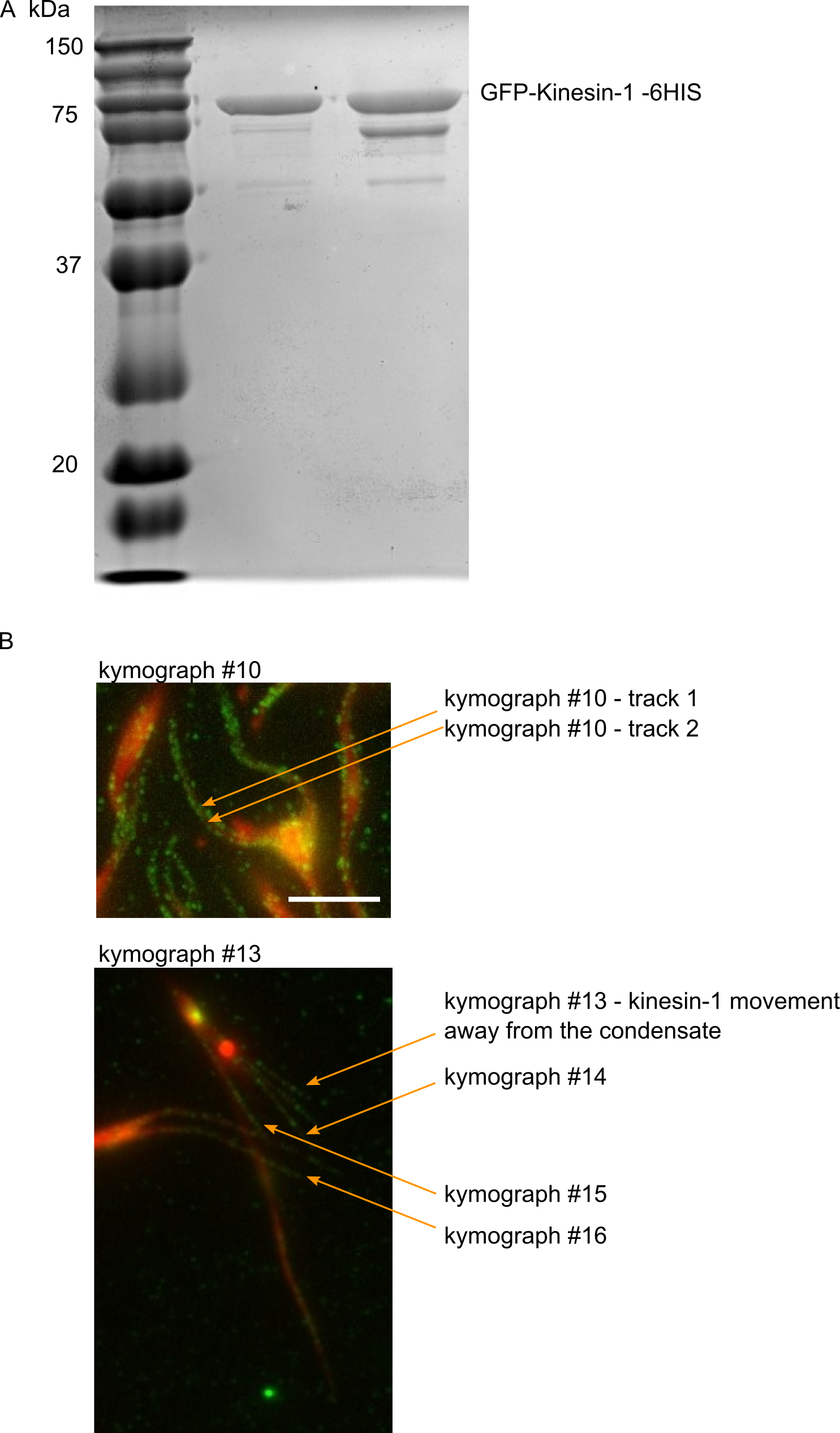


**Supplementary Figure 4. Kinesin-1-GFP purity and motorPAINT analysis** A. SDS-PAGE gel stained with Coomassie to show the composition and purity of the kinesin-1-GFP prep used in motor-PAINT experiment. B. Example of the large bundle (kymograph #10). that had two separable kinesin-1 tracks with opposite directions (toward and away from the condensate). We believe that these types of large MT bundles are formed by fusing already formed MT bundles of same orientation of MTs. Due to nature of TIRF, we may not observe the remaining part of the bundle that is above the TIRF acquisition plane. Different MT bundles may still be fused together in orientation that results in the overlapping kinesin tracks thus resulting in bundles classified as multidirectional. Note that the CPC condensate origin of the bundle may potentially be misidentified and bundle classified with away orientation (the bundle origin of kymograph #13 is less clear than the #14 - #16, and it may come from the condensate that is not visible in TIRF acquisition plane); scale bar = 5µm.

**Supplementary Figure 5**

**
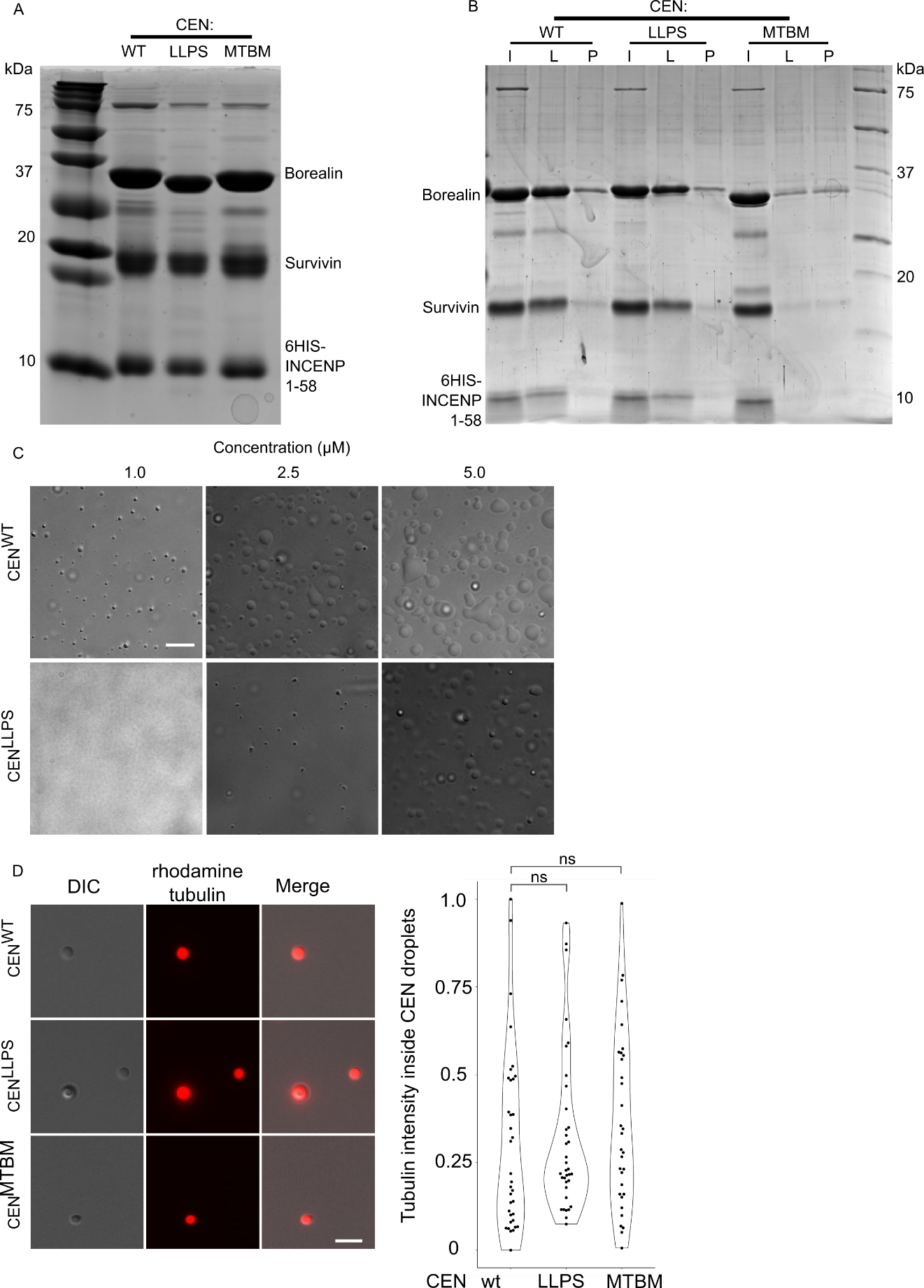
**

**Supplementary Figure 5. Purity of recombinant CEN-Borealin^wt^, CEN-Borealin^LLPS^, CEN-Borealin^MTBM^ protein preps**. A. SDS-PAGE gel stained with Coomassie to show components and purity of the CEN wild type and mutant preps used in MTs polymerization and bundling experiments. B. To verify that all components of the purified CEN complexes are retained in condensates, phase separation was induced by dilution into 500 mM NaCl + 7% PEG-3350. Condensed material was isolated by centrifugation, separated by SDS-PAGE, and stained with Coomassie (“Pellet” lanes). An equal mass of soluble protein was added directly to sample buffer (“Input lanes”) for comparison. C. The CEN^LLPS^ mutant is deficient in LLPS but still forms condensates. DIC images of CEN^WT^ or CEN^LLPS^ condensates in 150 mM NaCl at the indicated protein concentration. Scale bar: 10 µm. D. Images of rhodamine-labelled tubulin sequestered by CEN droplets. The plot shows quantification of tubulin intensity inside CEN droplets. CEN LLPS and MTBM droplets concentrate tubulin in the absence of GTP similarly to CEN WT. CEN-Borealin^wt^ n=(35 droplets); CEN-Borealin^LLPS^ n=(35 droplets); CEN-Borealin^MTBM^ n=(32 droplets). p-value^wt-LLPS^ = (0.11); p-value^wt-MTBM^ = (0.27); scale bar = 5µm. For statistical analysis Kolmogorov-Smirnov test was applied; * = p<0.05, ** = p < 0.01, *** = p<0.001

**Supplementary Figure 6**


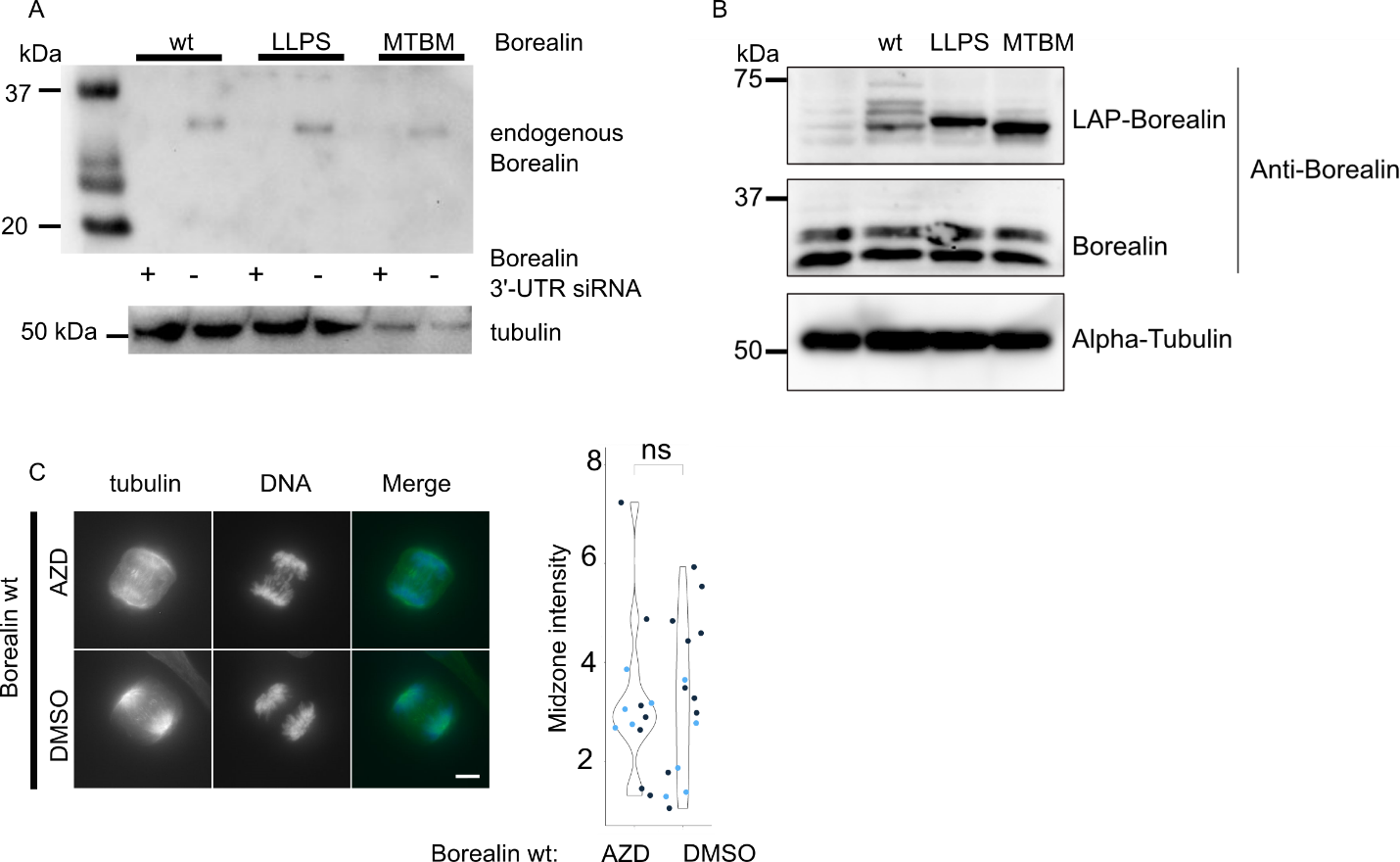


**Supplementary Figure 6**. **Western blot of Borealin siRNA knock-down and replacement and immunofluorescence analysis of Borealin depleted cells.** A. Western blot showing endogenous Borealin level in HeLa cells with or without treatment with Borealin 3`-UTR siRNA. B. Western blot showing Borealin levels in cells blocked in mitosis by nocodazole to show that the levels of the endogenous and exogenous proteins are similar. C. Midzone MTs levels in HeLa LAP-Borealin^wt^ cells treated with AZD1152-HQPA n=(10, 14) and DMSO n=(20, 10). Experiment was repeated twice; scale bar = 5µm. For statistical analysis Welch's t-test with Bonferroni correction was applied; p-value^AZD-DMSO^ = (0.9, 0.174). * = p<0.05, *** = p < 0.01, *** = p<0.001, ns = p>0.05.

**Supplementary data #7**

Uncropped version of Western blot in Figure 1 E

**
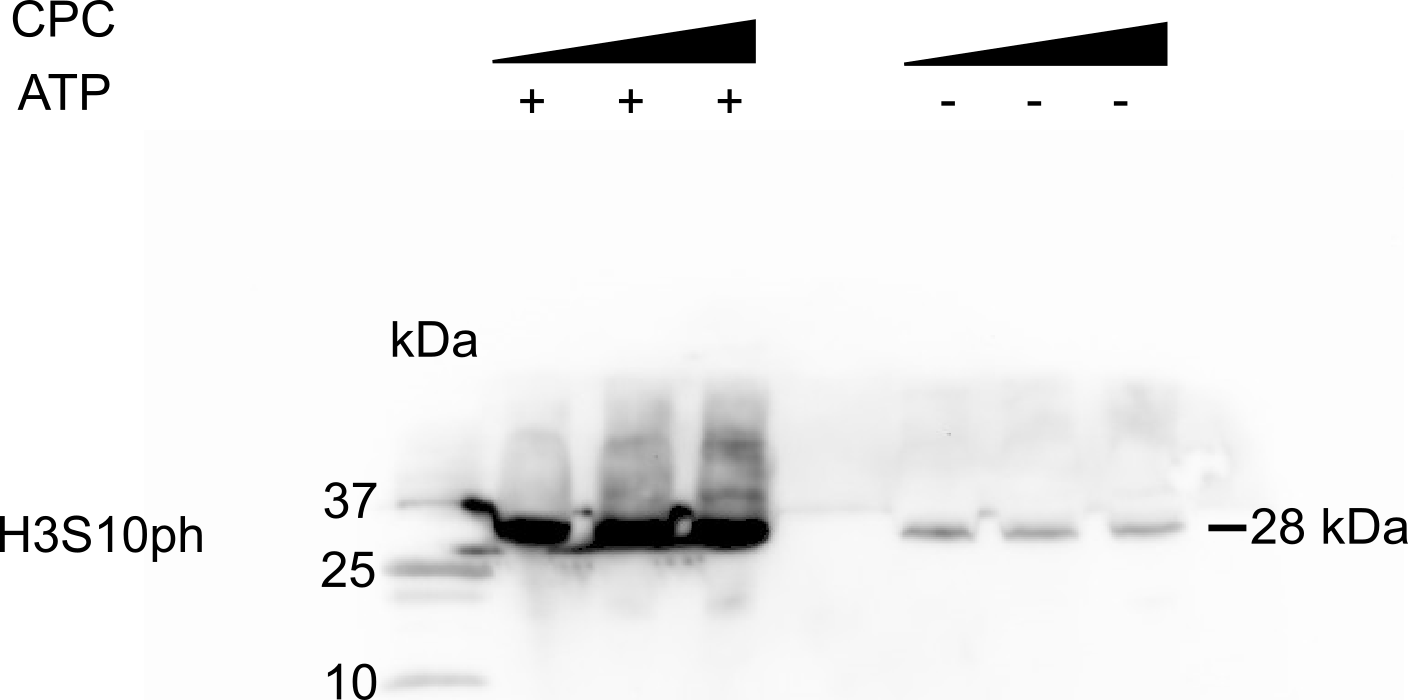
**

28 kDa is a molecular mass of GST tag plus the first 21 amino acids of histone H3.

Uncropped version of Coomassie stained gel in Supplementary Figure 1D

**
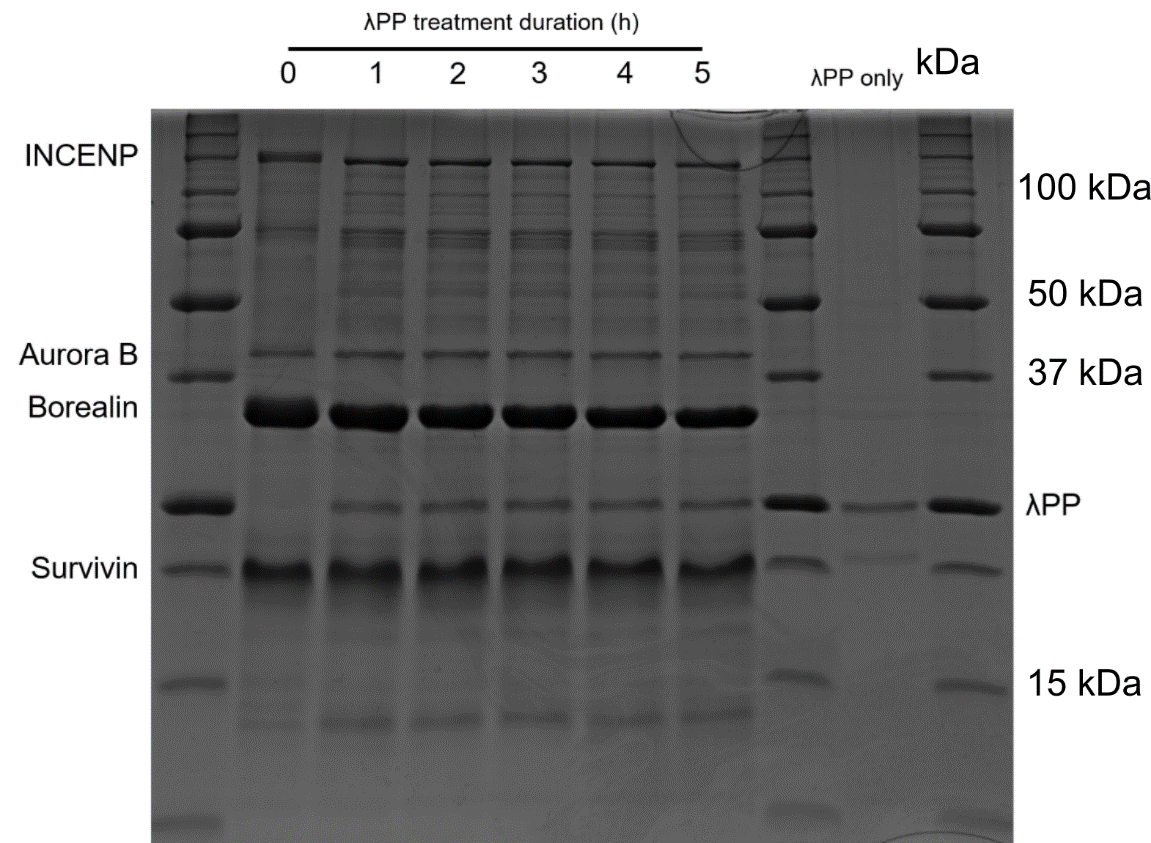
**

Uncropped version of Western blots in Supplementary Figure 6

| 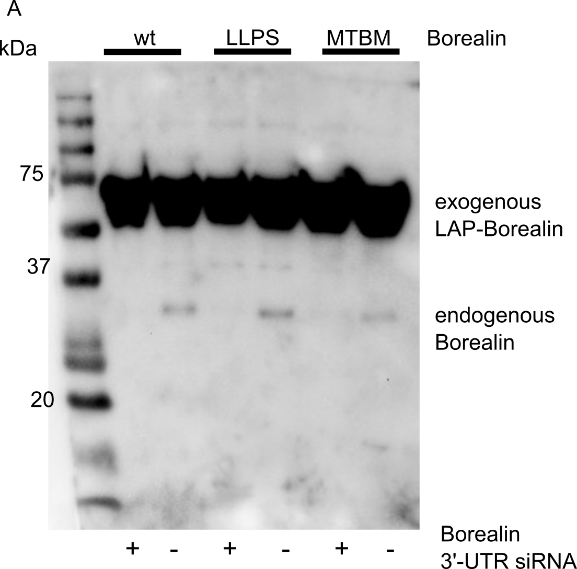 | 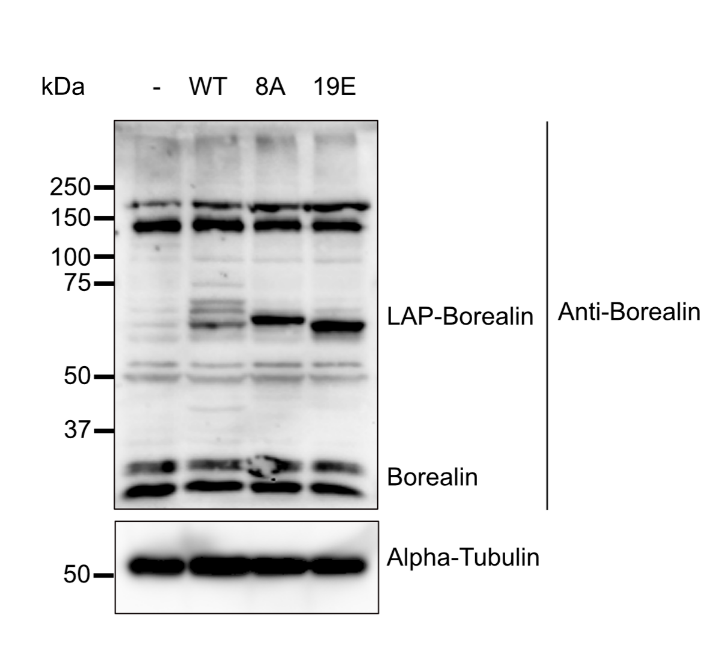 |
| --- | --- |

**Supplementary data #8**

Amino acid sequences of proteins used in the study

**xAuroraB (Δ30); UNP: Q6DE08**

MAYVSTFTTPSDNLLAQRTQLSRITPSASSSVPGRVAVSTEMPSQNTALAEMPKRKFTIDDFDIGRPLGKGKFGNVYLAREKQNKFIMALKVLFKSQLEKEGVEHQLRREIEIQSHLRHPNILRMYNYFHDRKRIYLMLEFAPRGELYKELQKHGRFDEQRSATFMEELADALHYCHERKVIHRDIKPENLLMGYKGELKIADFGWSVHAPSLRRRTMCGTLDYLPPEMIEGKTHDEKVDLWCAGVLCYEFLVGMPPFDSPSHTETHRRIVNVDLKFPPFLSDGSKDLISKLLRYHPPQRLPLKGVMEHPWVKANSRRVLPPVYQSTQSK

**xINCENP; UNP:O13024**

MNDAECLSHLLQVCARKTEEFVRTLDSKHMVWLLEIEEEARKMFSSDFNAEPELMPKTPSQKRRRKKRTSILPDENRDPSGRRISRRQSNASWSSSVRRLSVRNQNKANDDSIQEEPAQLKRMTRARAQASIKSTPVLETALPESPSQICQKNAQVKISEQERRSAEQKLIESDFELKTVPEITKDNVSETVNSAVPAVPVTPENKSRAAGKLKIAASSTPEQKAEMVDLTCESPRPANEQQLNLSNQSATPTGSKSDRRSVRRSLVVRKSSSRRASLASQFSLASKRESMTREAVRKSIRQSISQKKAAMEISSTSSQRSYQSSIEMVDDEITIKIRPETVPSETVSEEAPAAESPRRSLRSRAFKKIAISNLPDSEEPPRKVTRQMVAGNAEPTPETTEDAQNIRRKSYKRAVDELSDDERPSEEERSPPRKKTPSPPCPPSKIVRPPPHMKSFLHTVQKNQLLMMTPGSIGKNIIMKSFIKRNTPLKTDPKTEEKERQRLDALRKKEEAELQRKQKIEEGKKRKQEELKVRREERLRKVLQARERVEQLEEEKKKKIEQKFAQIDEKSEKVREDRMAEEKAKKKMTAKKQEEVECRRKQEEEARRLKVKQMEEEERRHQELLQKKREEEELERQKKIAEAKRLAEQERERQLLAEKERLRAEREKERIEKEKALQLQRELERAAQEKEQQRREAEERKKREQQERLEQERLRKEQEAKRLQEEEQRKAKEQAAVAASAPVMNVTVDMQNSPACESYEMTPKSCKVPSVKVNEDNYGMDLNSDDSTDDESQPRKPIPAWASGNLLTQAIRQQYYKPIDVDRMYGTIDSPKLEELFNKSKPRYFKRTSSAVWHSPPLSSNRHHLAVGYGLKY

**xSurvivin; UNP:** **Q8JGN5**

MYSAKNRFVQAVQRLQDFKNMYDYDARLATFADWPFTENCKCTPESMAKAGFVHCPTENEPDVACCFFCLKELEGWEPDDDPWTEHSKRSANCGFLSLTKCVNDLTMEGFLRLEGDRIKSFYRKFSTVVLQYVEEEMTAATKRLLEYFSNQHHCSIDLDH

**xDasra; UNP: Q4V7H8**

MPPKRNRNRLGTRGEGSGDSGVGMFERNDAVQEHKKEKIRLFMQDFVQQGKDRLAELKKDLESLSTTADKALEVELLKMPLAIRHMKVQDYLSLMGGDKSAVAAAAVKLDCSVDELSEPKLVRKNSKKVKVTTNVEYQDDVRTKVMTTSAKNRTVQKVPKSKSMLSLTGKNGKKTTALTRSVSATPLDKASKKLLVTNSSSKPAQRSSRTAMTPLTRSARSDTMFTFGDGAFLDEGVPFVKIPLADGKTVFSAGDDLDSLNVELLRGDTVQHIHNLVGQLTSLCAKASIQHHGKTL

**hINCENP (1-58aa); UNP:Q9NQS7**

MGTTAPGPIHLLELCDQKLMEFLCNMDNKDLVWLEEIQEEAERMFTREFSKEPELMPK

**hSurvivin; UNP: O15392**

MGAPTLPPAWQPFLKDHRISTFKNWPFLEGCACTPERMAEAGFIHCPTENEPDLAQCFFCFKELEGWEPDDDPIEEHKKHSSGCAFLSVKKQFEELTLGEFLKLDRERAKNKIAKETNNKKKEFEETAKKVRRAIEQLAAMD

**hBorealin; UNP:Q53HL2**

MAPRKGSSRVAKTNSLRRRKLASFLKDFDREVEIRIKQIESDRQNLLKEVDNLYNIEILRLPKALREMNWLDYFALGGNKQALEEAATADLDITEINKLTAEAIQTPLKSAKTRKVIQVDEMIVEEEEEEENERKNLQTARVKRCPPSKKRTQSIQGKGKGKRSSRANTVTPAVGRLEVSMVKPTPGLTPRFDSRVFKTPGLRTPAAGERIYNISGNGSPLADSKEIFLTVPVGGGESLRLLASDLQRHSIAQLDPEALGNIKKLSNRLAQICSSIRTHK

**hBorealin LLPS; UNP:Q53HL2 with the following mutations: R141A, K143A, R144A, K149A, K150A, R151A, K158A, K160A.**

MAPRKGSSRVAKTNSLRRRKLASFLKDFDREVEIRIKQIESDRQNLLKEVDNLYNIEILRLPKALREMNWLDYFALGGNKQALEEAATADLDITEINKLTAEAIQTPLKSAKTRKVIQVDEMIVEEEEEEENERKNLQTAAVAACPPSAAATQSIQGAGAGKRSSRANTVTPAVGRLEVSMVKPTPGLTPRFDSRVFKTPGXRTPAAGERIYNISGNGSPLADSKEIFLTVPVGGGESLRLLASDLQRHSIAQLDPEALGNIKKLSNRLAQICSSIRTHK

**hBorealin MTBM; UNP:Q53HL2 with the following mutations: R17E, R19E, K20E**

MAPRKGSSRVAKTNSLEREELASFLKDFDREVEIRIKQIESDRQNLLKEVDNLYNIEILRLPKALREMNWLDYFALGGNKQALEEAATADLDITEINKLTAEAIQTPLKSAKTRKVIQVDEMIVEEEEEEENERKNLQTARVKRCPPSKKRTQSIQGKGKGKRSSRANTVTPAVGRLEVSMVKPTPGLTPRFDSRVFKTPGLRTPAAGERIYNISGNGSPLADSKEIFLTVPVGGGESLRLLASDLQRHSIAQLDPEALGNIKKLSNRLAQICSSIRTHK

**Supplementary data #9**

Supplementary Table 1. Non-CPC proteins detected by MS analysis

| **Accession** | **Description** | **Coverage [%]** | **# Peptides** | **# PSMs** | **# Unique Peptides** | **# AAs** | **MW [kDa]** | **calc. pI** | **# Peptides (by Search Engine)** | **# Peptides (by Search Engine)** | **MS Abundance: Sample 1** | **MS Abundance: Sample 2** |
| --- | --- | --- | --- | --- | --- | --- | --- | --- | --- | --- | --- | --- |
| ALBU_BOVIN | (Common contaminant protein) | 95 | 232 | 11572 | 232 | 607 | 69.2 | 6.18 | 149 | 83 | 1.487E+11 | 59721472417 |
| A0A140N587 | Bifunctional polymyxin resistance protein ArnA OS=Escherichia coli (strain B / BL21-DE3) OX=469008 GN=arnA PE=3 SV=1 | 71 | 35 | 113 | 35 | 660 | 74.2 | 6.87 | 34 | 1 | 69172835.2 | 38864947.62 |
| K2C1_HUMAN | (Common contaminant protein) | 58 | 38 | 109 | 38 | 643 | 65.8 | 8.12 | 30 | 8 | 78529726.5 | 202090917.6 |
| A0A140NDX4 | Dihydrolipoyllysine-residue succinyltransferase component of 2-oxoglutarate dehydrogenase complex OS=Escherichia coli (strain B / BL21-DE3) OX=469008 GN=ECBD_2934 PE=3 SV=1 | 70 | 32 | 83 | 32 | 405 | 44 | 5.81 | 26 | 6 | 113255600 | 53322135.11 |
| A0A140NAY3 | Histidine biosynthesis bifunctional protein HisB OS=Escherichia coli (strain B / BL21-DE3) OX=469008 GN=hisB PE=3 SV=1 | 63 | 22 | 79 | 22 | 355 | 40.2 | 6.18 | 21 | 1 | 87755467.3 | 53812753.86 |
| K1C10_HUMAN | (Common contaminant protein) | 49 | 30 | 79 | 30 | 593 | 59.5 | 5.21 | 25 | 5 | 57626162.5 | 75205630.55 |
| K1C9_HUMAN | (Common contaminant protein) | 58 | 26 | 63 | 26 | 623 | 62.1 | 5.3 | 22 | 4 | 26037442.9 | 79199189.3 |
| A0A140NHM8 | Soluble pyridine nucleotide transhydrogenase OS=Escherichia coli (strain B / BL21-DE3) OX=469008 GN=sthA PE=3 SV=1 | 65 | 19 | 42 | 19 | 466 | 51.5 | 6.57 | 18 | 1 | 32279265.7 | 30145680.14 |
| A0A140NE66 | Oxoglutarate dehydrogenase (succinyl-transferring) OS=Escherichia coli (strain B / BL21-DE3) OX=469008 GN=ECBD_2935 PE=4 SV=1 | 33 | 20 | 39 | 20 | 933 | 105 | 6.49 | 19 | 1 | 22991047.5 | 10355246.2 |
| K22E_HUMAN | (Common contaminant protein) | 43 | 21 | 44 | 21 | 645 | 65.8 | 8 | 18 | 3 | 22132920.5 | 27931835.52 |
| A0A140N9J4 | Diamine N-acetyltransferase OS=Escherichia coli (strain B / BL21-DE3) OX=469008 GN=ECBD_2062 PE=4 SV=1 | 72 | 12 | 35 | 12 | 186 | 21.9 | 6.68 | 11 | 1 | 57700326.7 | 31643345.2 |
| A0A140N6V1 | Peptidyl-prolyl cis-trans isomerase OS=Escherichia coli (strain B / BL21-DE3) OX=469008 GN=ECBD_0400 PE=3 SV=1 | 71 | 12 | 53 | 12 | 196 | 20.8 | 5.05 | 8 | 4 | 65596019.3 | 143757636.4 |
| A0A140NGK1 | RNA-binding protein Hfq OS=Escherichia coli (strain B / BL21-DE3) OX=469008 GN=hfq PE=1 SV=1 | 99 | 10 | 100 | 10 | 102 | 11.2 | 7.65 | 10 |  | 878339757 | 443086525.7 |
| A0A140N5F4 | Polyribonucleotide nucleotidyltransferase OS=Escherichia coli (strain B / BL21-DE3) OX=469008 GN=pnp PE=3 SV=1 | 30 | 17 | 20 | 17 | 711 | 77.1 | 5.21 | 17 |  | 8732493.02 | 2186967.594 |
| A0A140NAH0 | Fructose-bisphosphate aldolase OS=Escherichia coli (strain B / BL21-DE3) OX=469008 GN=ECBD_1560 PE=4 SV=1 | 59 | 12 | 22 | 12 | 350 | 38.1 | 6.73 | 12 |  | 19223305.3 | 9298019.328 |
| TRYP_PIG | (Common contaminant protein) | 25 | 6 | 114 | 6 | 231 | 24.4 | 7.18 | 6 |  | 1396800417 | 837784449.9 |
| A0A140NE25 | Glutamine--fructose-6-phosphate aminotransferase [isomerizing] OS=Escherichia coli (strain B / BL21-DE3) OX=469008 GN=glmS PE=3 SV=1 | 33 | 11 | 18 | 11 | 609 | 66.9 | 5.87 | 11 |  | 3598601.85 | 3077437.242 |
| A0A140NDE3 | Outer membrane protein A OS=Escherichia coli (strain B / BL21-DE3) OX=469008 GN=ompA PE=3 SV=1 | 39 | 9 | 16 | 9 | 346 | 37.2 | 6.42 | 9 |  | 8889304.38 | 6614833.031 |
| A0A140NET2 | Trigger factor OS=Escherichia coli (strain B / BL21-DE3) OX=469008 GN=tig PE=3 SV=1 | 20 | 7 | 11 | 7 | 432 | 48.2 | 4.88 | 7 |  | 1871347.06 | 784840.9844 |
| A0A140NAA6 | Uncharacterized protein OS=Escherichia coli (strain B / BL21-DE3) OX=469008 GN=ECBD_1392 PE=3 SV=1 | 50 | 5 | 13 | 5 | 101 | 11.3 | 5.57 | 4 | 1 | 5119901.91 | 593662.1484 |
| A0A140NBF7 | Catalase OS=Escherichia coli (strain B / BL21-DE3) OX=469008 GN=ECBD_1913 PE=3 SV=1 | 19 | 8 | 11 | 8 | 753 | 84.1 | 5.82 | 7 | 1 | 1094285.16 | 2439913.609 |
| A0A140N7Y1 | Uncharacterized protein OS=Escherichia coli (strain B / BL21-DE3) OX=469008 GN=ECBD_0641 PE=3 SV=1 | 23 | 2 | 7 | 2 | 101 | 11 | 9.09 | 2 |  | 18315335.2 | 17749901.46 |
| A0A140ND72 | ATP synthase subunit alpha OS=Escherichia coli (strain B / BL21-DE3) OX=469008 GN=atpA PE=3 SV=1 | 16 | 5 | 6 | 5 | 513 | 55.2 | 6.13 | 5 |  | 5061650.5 | 679598.4688 |
| A0A140NH65 | 60 kDa chaperonin OS=Escherichia coli (strain B / BL21-DE3) OX=469008 GN=groL PE=3 SV=1 | 19 | 5 | 7 | 5 | 548 | 57.3 | 4.94 | 5 |  |  | 198766.9063 |
| A0A140N3D6 | Transcriptional regulator, Crp/Fnr family OS=Escherichia coli (strain B / BL21-DE3) OX=469008 GN=ECBD_0391 PE=4 SV=1 | 20 | 3 | 4 | 3 | 210 | 23.6 | 8.25 | 3 |  | 1591266 | 1170007.578 |
| A0A140SS84 | Acetylornithine deacetylase OS=Escherichia coli (strain B / BL21-DE3) OX=469008 GN=argE PE=3 SV=1 | 24 | 3 | 5 | 3 | 383 | 42.3 | 5.9 | 3 |  | 732774.344 | 470628.8594 |
| A0A140NFV3 | Chaperone protein DnaK OS=Escherichia coli (strain B / BL21-DE3) OX=469008 GN=dnaK PE=2 SV=1 | 14 | 5 | 7 | 5 | 638 | 69.1 | 4.97 | 5 |  | 981092.391 | 413079.9297 |
| A0A140SSB2 | Transcriptional regulator, LysR family OS=Escherichia coli (strain B / BL21-DE3) OX=469008 GN=ECBD_4208 PE=3 SV=1 | 11 | 3 | 5 | 3 | 298 | 33.4 | 6.95 | 3 |  | 1428121.44 | 879002.7188 |
| A0A140N8K1 | Transcriptional regulator, LysR family OS=Escherichia coli (strain B / BL21-DE3) OX=469008 GN=ECBD_1501 PE=3 SV=1 | 18 | 3 | 5 | 3 | 293 | 32.7 | 6.54 | 3 |  | 4263647.57 | 1256561.348 |
| A0A140NHS0 | ATP synthase subunit beta OS=Escherichia coli (strain B / BL21-DE3) OX=469008 GN=atpD PE=3 SV=1 | 14 | 4 | 4 | 4 | 460 | 50.3 | 5.01 | 4 |  | 248639.344 | 259255.625 |
| A0A140N7J1 | 50S ribosomal protein L2 OS=Escherichia coli (strain B / BL21-DE3) OX=469008 GN=rplB PE=3 SV=1 | 12 | 3 | 4 | 3 | 273 | 29.8 | 10.93 | 3 |  | 1925480.69 | 146263.2188 |
| A0A140NFZ9 | Chaperone protein DnaJ OS=Escherichia coli (strain B / BL21-DE3) OX=469008 GN=dnaJ PE=3 SV=1 | 14 | 2 | 3 | 2 | 376 | 41 | 7.66 | 2 |  | 1124020.07 |  |
| A0A140NA87 | Diguanylate cyclase OS=Escherichia coli (strain B / BL21-DE3) OX=469008 GN=ECBD_2104 PE=4 SV=1 | 7 | 2 | 2 | 2 | 296 | 33.9 | 5.95 | 2 |  | 266417.656 | 114940.0469 |
| A0A140ND04 | Nuclease SbcCD subunit C OS=Escherichia coli (strain B / BL21-DE3) OX=469008 GN=sbcC PE=3 SV=1 | 16 | 10 | 19 | 10 | 1048 | 118.6 | 5.68 | 4 | 6 | 762424.152 | 978305.3281 |
| A0A140NBT9 | Cell division protein FtsA OS=Escherichia coli (strain B / BL21-DE3) OX=469008 GN=ftsA PE=3 SV=1 | 27 | 5 | 6 | 5 | 420 | 45.3 | 6.24 | 5 |  | 1261760.18 | 913097.4189 |
| A0A140NA80 | Succinate dehydrogenase flavoprotein subunit OS=Escherichia coli (strain B / BL21-DE3) OX=469008 GN=ECBD_2937 PE=3 SV=1 | 11 | 4 | 6 | 4 | 588 | 64.4 | 6.27 | 4 |  | 583434 | 341190.7188 |
| K1C15_SHEEP | (Common contaminant protein) | 4 | 2 | 3 | 2 | 453 | 48.7 | 4.79 | 2 |  |  | 16938123.25 |
| A0A140NCI6 | Elongation factor Tu OS=Escherichia coli (strain B / BL21-DE3) OX=469008 GN=tuf PE=3 SV=1 | 13 | 3 | 5 | 3 | 394 | 43.3 | 5.45 | 3 |  | 422899.724 | 311741.8379 |
| A0A140N6W0 | Elongation factor Tu OS=Escherichia coli (strain B / BL21-DE3) OX=469008 GN=tuf PE=1 SV=1 | 13 | 3 | 5 | 3 | 394 | 43.3 | 5.45 | 3 |  | 422899.724 | 311741.8379 |
| ALBU_HUMAN | (Common contaminant protein) | 16 | 6 | 20 | 6 | 609 | 69.3 | 6.28 | 4 | 2 | 37481082.7 | 27172856.59 |
| A0A140NAB3 | Diguanylate cyclase with PAS/PAC sensor OS=Escherichia coli (strain B / BL21-DE3) OX=469008 GN=ECBD_2276 PE=4 SV=1 | 9 | 2 | 3 | 2 | 410 | 46.4 | 6.8 | 2 |  | 660413.578 | 634084.8125 |
| A0A140N7A6 | Uncharacterized protein OS=Escherichia coli (strain B / BL21-DE3) OX=469008 GN=ECBD_1047 PE=3 SV=1 | 30 | 2 | 6 | 2 | 109 | 11.8 | 8.54 | 2 |  | 747440.906 | 139969.4648 |
| A0A140N8D7 | Phosphomethylpyrimidine kinase OS=Escherichia coli (strain B / BL21-DE3) OX=469008 GN=ECBD_1554 PE=4 SV=1 | 16 | 2 | 2 | 2 | 266 | 28.7 | 6.15 | 2 |  | 643600.281 | 281809.7109 |
| A0A140N812 | Transcriptional regulator, PadR-like family OS=Escherichia coli (strain B / BL21-DE3) OX=469008 GN=ECBD_0671 PE=4 SV=1 | 8 | 2 | 3 | 2 | 207 | 23.4 | 6.74 | 2 |  | 573190.047 | 162190.1211 |
| A0A140N689 | NAD kinase OS=Escherichia coli (strain B / BL21-DE3) OX=469008 GN=nadK PE=3 SV=1 | 11 | 2 | 10 | 2 | 292 | 32.5 | 6.79 | 2 |  | 3506729.49 | 17906473.43 |
| A0A140N7T0 | Luciferase-like monooxygenase OS=Escherichia coli (strain B / BL21-DE3) OX=469008 GN=ECBD_0580 PE=4 SV=1 | 11 | 2 | 2 | 2 | 335 | 37.2 | 6.6 | 2 |  | 2833069.23 | 249679.2891 |
| A0A140N5C4 | Uncharacterized protein OS=Escherichia coli (strain B / BL21-DE3) OX=469008 GN=ECBD_0739 PE=4 SV=1 | 17 | 2 | 2 | 2 | 95 | 11 | 9.13 | 2 |  | 104953.07 | 66489.60938 |
| A0A140NFT6 | ATP synthase subunit delta OS=Escherichia coli (strain B / BL21-DE3) OX=469008 GN=atpH PE=3 SV=1 | 13 | 2 | 3 | 2 | 177 | 19.3 | 5.02 | 2 |  | 553007 | 3501433.063 |
| A0A140NCV3 | PpiC-type peptidyl-prolyl cis-trans isomerase OS=Escherichia coli (strain B / BL21-DE3) OX=469008 GN=ECBD_3214 PE=4 SV=1 | 4 | 2 | 3 | 2 | 623 | 68.1 | 5.05 |  | 2 |  |  |
| P0C1U8 | Glu-C Glutamyl endopeptidase OS=Staphylococcus aureus GN=sspA PE=1 SV=1(Common contaminant protein) | 67 | 29 | 295 | 29 | 336 | 36.3 | 5.19 |  | 29 | 7602393.69 | 13148951.81 |
