# Supplementary figures and images for: Chromosomal passenger complex condensates generate parallel microtubule bundles in vitro
Source: J Biol Chem. 2024 Jan 23;300(3):105669. doi: 10.1016/j.jbc.2024.105669 (PMC10876603; doi:10.1016/j.jbc.2024.105669)

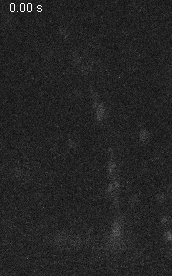

Supplement: Movie S1 [file mmc2.gif]
